# The Five Marks of the Mental

**DOI:** 10.3389/fpsyg.2017.01084

**Published:** 2017-07-07

**Authors:** Tuomas K. Pernu

**Affiliations:** ^1^Department of Philosophy, King’s College LondonLondon, United Kingdom; ^2^Division of Physiology and Neuroscience, Department of Biosciences, University of HelsinkiHelsinki, Finland

**Keywords:** access consciousness, folk psychology, free will, mind-body problem, intentionality, normativity, phenomenal consciousness, teleology

## Abstract

The mental realm seems different to the physical realm; the mental is thought to be dependent on, yet distinct from the physical. But how, exactly, are the two realms supposed to be different, and what, exactly, creates the seemingly insurmountable juxtaposition between the mental and the physical? This review identifies and discusses five marks of the mental, features that set characteristically mental phenomena apart from the characteristically physical phenomena. These five marks (intentionality, consciousness, free will, teleology, and normativity) are not presented as a set of features that define mentality. Rather, each of them is something we seem to associate with phenomena we consider mental, and each of them seems to be in tension with the physical view of reality in its own particular way. It is thus suggested how there is no single mind-body problem, but a set of distinct but interconnected problems. Each of these separate problems is analyzed, and their differences, similarities and connections are identified. This provides a useful basis for future theoretical work on psychology and philosophy of mind, that until now has too often suffered from unclarities, inadequacies, and conflations.

## Introduction

The mental, or the psychological realm is typically conceived to be different from the physical, or the biological and neural realm. The mental is thought to be dependent on, yet distinct from the physical. The two are not identical, and the former is not reducible to the latter – according to many – yet they are coordinated with each other, and even in interaction with each other, or at least so it appears. Many theoretical problems arise from this: the issue of how, or indeed whether, the mind and the body can interact, debates on whether the mental is ultimately reducible to the physical, claims that there is no genuine science of psychology, autonomous from the more basic – more real – sciences of biology, neuroscience, and physics, and, most distressingly, doubts about there being no such thing as real psychological agency and free will.

It thus appears that there is no single mind-body problem, but a plethora of profound and complex issues, tightly intertwined. There is, however, one common thread in all these issues: the assumption that there is something that is characteristically mental, a feature, or a set of features, that makes it difficult for us to identify the mental with the physical, or conjoin the two. So the question arises: how, exactly, are the two realms different, and what are the features and issues that seem to set characteristically mental phenomena irrevocably apart from the characteristically physical phenomena – what are the *marks of the mental*?

The following identifies and analyses five features that we closely associate with characteristically mental phenomena. The intention of this analysis is not to present an individually necessary but jointly sufficient set of features that define mentality. Rather, each of these features is something we seem to associate with phenomena we consider mental, and each of them seems to be in tension with the physical view of reality in their own particular way. Each of these features thus creates its own particular mind-body problem, that is largely distinct from, although also intimately connected to, the other problems.

We are thus faced here with two tasks: first, to describe and analyze the five characteristically mental features, and second, to identify and unpack their connections and interdependencies. The five marks of the mental discussed here are: intentionality, consciousness, free will, teleology, and normativity. Only the first two of these are typically identified and discussed, with often the first one being granted the title of the mark of the mental (e.g., Place, 1996; Crane, 1998a,b, 2009; Horgan and Kriegel, 2008; Tartaglia, 2008; Voltolini, 2013; Jacob, 2014; Neander, 2017). One central aim of the discussion to follow is thus to show, or at least to suggest, that the traditional discussion has been radically incomplete. However, the issue of whether this incompleteness is merely superficial is left largely unaddressed. The five marks discussed here are to be understood as signs or symptoms of mentality, and the question of whether these marks can ultimately be reduced to one or two more primitive marks will need to be answered separately.

The issues on focus here are profound and complicated, with each of them having long and tangled histories. It is not possible to do justice to all the nuances of the issues in the confines of this essay; many central philosophical problems related to each of the issues will have to be left unaddressed. Listing every issue and discussing all the fine grained problems is not the aim here, however, although deciphering such details is admittedly ultimately relevant. The main goal here is to outline a bigger picture – to take a step back and have a view from a distance, if you will – and show how the single notion of “the mental” will actually divide into a number of sub-notions, and how these “parts of the mental” can be linked to each other. In other words, the aim here is not to provide a complete treatment of each of the marks of the mental, but focus on the characteristic aspects of each of them, as marks of the mental, and sketch their connections. Details are not irrelevant to such a project, of course, but providing a consistent overall view is equally important.

## The Mind Problem, The Body Problem, And the Mind-Body Problem

Let us start by getting a clearer understanding of what is at issue here. The term “mark of the mental” (or “marks of the mental”) is often taken to denote the necessary and sufficient criterion (or a set of criteria) that unequivocally defines the essence of mentality: things (phenomena or processes) that have the mark, or the appropriate set of them, are mental, things don’t, aren’t. The issue of what is mental and what is not is clear-cut, in principle at least.

This is not the view advocated here. The marks of the mental are here presented as points of tension, features that create a distinctive contrast to the physical. Consequently, the following is not only an analysis of the marks of the mental, it is also, indirectly, an analysis of the marks of the physical. The two realms are, in essence, inter-definable. This is not a superficial terminological issue, but a deep metaphysical one: the notions appear in our discourse only as contrastive signposts, and in using one, one is always referring to the other as well, at least tacitly. There is no “mentality” or “physicality” over and above the distinct contrastive features that we associate with the two realms. There are only pieces or aspects of each, conceptually linked to their counterparts.

Consider an analogy: what are the “marks of the living”? First, as in the case of mentality, there is no well-defined set of features that all and only animate entities have and the inanimate lack. The features that are typically shared by phenomena and processes we recognize as living are such as growth and change in time (at various scales), response to stimuli and long-term adaptation, internal functional and hierarchical organization, the presence of homeostatic mechanisms and metabolism, and the ability to reproduce and pass genetic information across generations. Second, these “marks of the living” are essentially contrastive in their nature: they are features of the animate only in contrast to the features that we associate with the inanimate.

The often overlooked result of this is that the mind-body problem can actually be broken down to two separate, but interconnected problems: the mind problem and the body problem. It is typical to think that it is the mind part of this problem that is somehow more vexing or intriguing, and thus in demand of more philosophical attention. But it is by no means clear what the “body” in the mind-body problem ultimately amounts to (Montero, 1999). For example, there is the well-known dilemma, originally formulated by Hempel (), of how to define the “physical” in a non-question-begging way: either the term refers to entities recognized as real by the current physics, in which case we can rest assured that physicalism is downright false (for our current understanding of physics is incomplete, and our views will change and theories will get discarded), or the term refers to entities of some ideal complete physics, in which case the content of the notion is wholly arbitrary (for no one knows what such a physics will look like and what sort of entities it might posit).

The “mental” and the “physical” are thus bound to go hand-in-hand, and in addressing one aspect of the mind-body problem one is necessarily also addressing the other. However, this does not preclude specific ways of emphasizing the issues. Since the focus is here on the marks of the mental, rather than on the marks of the physical, the following will thus look at the mind-body problem from the former perspective. In other words, it is the mental realm that is seen as particularly anomalous in contrast to the physical realm (based on our current understanding). The marks discussed here are specific anomalous features of mentality that seem to shun physical explanation.

It is worth stressing that the issue here is different to, and wholly independent of, the question of whether the mental is ultimately identical with or reducible to the physical, or whether some sort of dualism is in fact the right metaphysical view to adopt. In particular, it misses the point to insist that this discussion presupposes some sort of Cartesian dualism, and that simply renouncing such dualism would immediately resolve all the problems surrounding topic. The mental and the physical are in apparent conceptual tension with each other. The tension will not disappear by fiat. To make progress, one needs to identify the elements of the tension and address each of them. Maybe the mental and the physical are identical, or the former is reducible to the latter, or maybe dualism prevails. But whichever view we are to adopt the decision should be based on a careful analysis on what is really at issue – arriving at the right diagnosis is always the first step toward an effective remedy.

## Intentionality

Intentionality is the traditional and most widely recognized mark of the mental. Indeed, to many, it is the only defining feature of mentality. The idea that intentionality is both necessary and sufficient for a phenomenon to be called mental is known by the name of *Brentano’s Thesis*. Here is a revealing, classic quote from Brentano (1874/1995):

“Every mental phenomenon is characterized by what the Scholastics of the Middle Ages called the intentional (or mental) inexistence of an object, and what we might call, though not wholly unambiguously, reference to a content, direction toward an object (which is not to be understood here as meaning a thing), or immanent objectivity. Every mental phenomenon includes something as object within itself, although they do not do so in the same way. In presentation, something is presented, in judgment something is affirmed or denied, in love loved, in hate hated, in desire desired, and so on.

    This intentional inexistence is characteristic exclusively of mental phenomena. No physical phenomenon exhibits anything like it. We can, therefore, define mental phenomena by saying that they are those phenomena which contain an object intentionally within themselves (Brentano, 1874/1995, p. 68).

What is important about this quote is that not only does it introduce the idea that it is exactly intentionality that is supposed to set mental phenomena apart from physical phenomena, but that it does so in various different ways. Intentionality itself thus divides into sub-marks of the mental.

The most prominent feature of intentionality is *aboutness*: the fact that we ascribe semantic or representational content to most of our conscious mental states. The peculiar thing here is the *relational* character of intentional mental states, their “direction toward an object.” We think, desire, love, hate, fear things outside of ourselves, both our bodies and our minds. The objects of our minds can, at least in most typical cases, be stated in propositional terms: we *think that* it is going to rain, but later we *see that* it doesn’t. The most central feature of intentionality is that most of our actions can be explained by combining propositional attitudes to propositional content: the fact that I took an umbrella with me in the morning is explained by my belief that it was going to rain; it is my belief, and the specific content it has, that explains why I behaved the way I did. This is a very intuitive, deeply entrenched, folk psychological way of making sense of our behavior. It is well-nigh impossible to think that there would be anything wrong with it, at least not radically, as indicated by this other, almost as often quoted, but more recent passage:

“[I]f it isn’t literally true that my wanting is causally responsible for my reaching, and my itching is causally responsible for my scratching, and my believing is causally responsible for my saying.... If none of that is literally true, then practically everything I believe about anything is false and it’s the end of the world.” (Fodor, 1989, p. 77.)

Another characteristic feature of intentionality needs to be separately acknowledged. This is the feature that is Brentano’s main focus in the quote above: the fact that the intentional content, or the objects of our mental states are characteristically non-veridical or inexistent (cf. also Meinong, 1899; Husserl, 1901). Now, it is not essential whether “intentional inexistence” is a necessary feature of all mental states, or even the intentional subset of them, or a merely contingent one. What’s essential is that many of our mental states are clearly about things that don’t exist. People think, believe, fear and wish, and even see and hear, things that are not real, and the fact that the content of these mental states is in this way non-veridical is not relevant to the role that these states play. As illusions, hallucinations, and delusions concretely show, it is often the non-veridical content of mental states that we need to refer to when explaining each other’s psyche and behavior.

So what, exactly, makes intentionality a mark of the mental then? What is the characteristically non-physical feature here that makes intentional mental states inexplicable – at least in any straightforward way – from a physical perspective? Intentional inexistence is one intuitively apparent way in which intentionality can seem anomalous to us (cf. Crane, 2001, 2012, 2013; Priest, 2005). The inexistence in question here is specifically physical, objective non-existence, not non-existence *tout court*, and the whole point in turning attention to this feature, and using this term, is to lead one to focus on the stark tension between physical non-existence on the one hand, and mental existence on the other; in other words, there is a sharp contrast between objective, physical *non*-existence and subjective, mental *in*existence. Illusions, hallucinations and delusions, and all other intentional entities, are psychologically real, but physically unreal. That is why intentional inexistence is difficult to reconcile with a wholly physical view on reality, and that is why it is a paradigmatic mark of the mental; mental entities and processes are within us – in our heads and in our minds – not out there, objectively in the world.

Although it is easy to hold intentional inexistence as a paradigmatic feature of the mental, it is worth noting that some recent developments seem to challenge such an idea. The fundamental thesis in the discussions surrounding the embodied cognition and extended mind hypotheses is the claim that the mind is *not* in the head, at least not in some notable parts – that it extends to the whole body, and even beyond it (e.g., Clark, 1997, 2001, 2003, 2008; Clark and Chalmers, 1998; Wilson, 2005; Logan, 2007; Menary, 2007, 2010; Chemero, 2009; Arnau et al., 2014). The idea of subjective, private intentional existence of mental entities and processes would thus become obsolete (cf. Malafouris, 2010, 2013). Note, however, that the extended mind hypothesis is not an eliminativist or a reductionist thesis, quite the contrary: the whole point is to expand the scope of mental notions. It would also be a mistake to conclude that the idea of intentional inexistence could now simply be dispensed with. If the extended mind hypothesis becomes generally accepted, it would clearly show that intentional inexistence is not a necessary mark of the mental. But such a result would not change the fact that intentional inexistence is a feature of many, if not most, of the phenomena we recognize as mental.

There are also other notable ways in which intentionality can appear anomalous to us, which relate more to the aboutness aspect of intentionality. Much of the past 40 years of philosophy of mind has been focusing on the issue of the semantic content of mental states – what is it, and how to account for it in perfectly physicalistic terms. The issue in focus can be seen to originate from the rejection of substance dualism: the mind is not viewed as a distinct entity separate from the body, subsisting in its own realm of reality, but as a cluster of mental faculties that are grounded in perfectly physical entities and processes. In other words, mentality is a function rather than a substance, and the seemingly insurmountable metaphysical disparity between the mind and the body can be seen to amount to nothing more peculiar than the difference between the software and hardware in a digital computer; the mind is something that simply does the computing on the basis of the input (stimuli), and then produces an output (behavior) (see **Box 1**).

**Box 1. Functional theory of mind in early imperial China.**

Understanding the mind as a function rather than as a substance has a long and intriguingly cross-cultural history. Here is an excerpt from a Chinese treatise *Shên-mieh lun* (“Essay on the extinction of the soul”) composed by a Confucian *Fan Chên* in the 500th century, portraying startling similarities to the contemporary discussion (from Balázs, 1964, p. 266):

(1) *Someone asked me:* You say the soul becomes extinguished. How do you know it becomes extinguished?
     *Answer:* The soul and the body are identical. Therefore while the body survives the soul survives, and when the body perishes the soul is extinguished.
(2) *Q.* “Body” refers to something that lacks consciousness, “soul” to something that has consciousness. Consciousness and lack of consciousness are two different things, therefore soul and body cannot reasonably be treated as one. I have never before heard it said that body and soul are identical.
     *A.* The body is the soul’s material basis; the soul is the functioning of the body. Consequently, since “body” refers to the material basis and soul to the functioning, body and soul cannot be regarded as separate.
(3) *Q.* But since admittedly, the soul is not the material basis, and the body not the functioning, where is the sense in saying that they cannot be regarded as separate?
     *A.* These are separate names referring to a single object.

So far so good. The age-old metaphysical puzzle on the relationship of the mind and the body seems to finally now be solved, and, most importantly, in a way that preserves a distinctive but perfectly natural role for the mind. However, a closer look reveals that something important seems to be left out: it is in the very idea of computation that it is a wholly syntactic process, whereas the hallmark of mental processes is that they are based on semantic content. The functionalist solution to the mind-body problem seems to leave the mind rather empty (e.g., Stich, 1983; Field, 2001).

There are many ways to make this problem more concrete. The fundamental issue to draw attention to is the relational character of mental states, the fact that they are about something – directed toward an object. I’m thinking about Donald Trump and you’re thinking about Donald Trump and the fact that we have the same person in mind explains our behavior (verbal discourse, let’s say) with respect to issues relating to American politics. But how can our thoughts be about something that is external to us? And even if they could, how could that play a role in bringing about our behavior?

There is thus a tension between internalist and externalist intuitions. On the one hand the contents of our minds are something very private and subjective, something within us – or so at least it very vividly seems to us. On the other hand the contents of our minds are about things other than ourselves, about entities and events that are both temporally and spatially located outside of us – or so at least it feels natural for us to think. The core of the problem is that to tackle the issue we cannot just simply move everything within us, or within our minds, as one might at first eagerly suggest: our minds, or at least their intentional content, were supposed to represent things outside themselves to enable us to coordinate ourselves with the environment and with each other – that is the whole point of having minds! It is simply a truism that representation is a relation between that which represents and that which is being represented. If our minds have representational content, then it rather trivially follows that our minds, or their contents at least, are necessarily relational, and therefore not within us. And if that is the case, then it seems difficult to understand how our minds, or their contents, could be in any way relevant to producing our behavior.

How all of this creates a stark juxtaposition between the mental and the physical becomes particularly clear when you consider the initially attractive idea that the mind operates on semantic representations grounded on brain, which in turn operates wholly syntactically. Perhaps the most well-known way to demonstrate the problem that arises from this is due to Searle (1980; Preston and Bishop, 2002). Suppose you don’t speak a word of Chinese. Imagine yourself now inside a closed room which contains a huge comprehensive rulebook connecting Chinese symbols to one another and describing how to respond with a certain symbol when presented with another. Suppose that there is a slot in the room through which you start receiving Chinese symbols. You turn to your book, look the symbol up, and respond by slipping another symbol back through the slot. Given that the rulebook is comprehensive, as was assumed, it would appear to any outside observer that you would be having a perfectly intelligible conversation and that you would be a fluent Chinese speaker. But of course you would not understand a word you are “saying.” All that you did was following the perfectly formal rules stated in the book. The meanings of the symbols, what they represent, would thus not seem to have any role to play in producing your behavior. Similarly, all that you say and all that you do is the result of perfectly formal processes run by your syntactic brain, and the representations in your mind have no part to play in that.

Another illustrative example is due to Dretske (1998, 2015). Consider a normal vending machine, operating on coins. It is natural for us to describe its behavior – the coordination between inputs and outputs – as if it is based on the value of the money that is being inserted into the machine: inserting a certain amount of money – which you can put together in various different ways by coins of different values – and pressing a certain button will give you a certain item in return. However, value is a relational, historical property: money has its value only if it has been produced the right way in a right place – counterfeit money has no value. But counterfeit coins are perfectly good currency for the vending machine. In fact, it is pretty clear that how the machine operates, and what it ejects, does not depend on the value of the coins inserted, but on their simple physical properties – size, shape, and weight. Similarly, the representational content of your mind plays no role in the inner-workings of your brain and body; your responses to the stimuli you receive are perfectly determined by physical features of the stimuli and your body.

This is not meant to be read as a demonstration of the utter insolubility of the problem of intentionality. There is no shortage of elaborate theories of mental content, and there is no end in sight to the debates raging between the internalists and externalists. These theories would have no audience, and these debates would not make sense, without a widely held consensus that progress on this issue is possible. But what all this does demonstrate, is that intentionality is a feature that keeps on escaping our attempts to understand it in purely physical terms. That is why it is a central mark of the mental.

## Consciousness

Consider next another paradigmatic feature of the mental, a feature that is perhaps even more familiar and tangible to us than intentionality: consciousness. Now, it is clear that intentionality and consciousness are connected in important ways, and often many various different discussions of the mind-body problem are linked to the issue of consciousness, one way or another. However, the point here is to draw attention to the problematic issues that are specifically due to consciousness, to identify them, and separate them from the other marks of the mental. A lot of confusion has been created by lumping together separate, fundamentally different issues as stemming from “the problem of consciousness.”

To zoom in on what’s at issue in here, it is useful to consider the by now familiar distinction between the so-called easy and hard problems of consciousness (Chalmers, 1995, 1996). The “easy problems” are really not that easy, of course, and perhaps it would be more useful to talk about tractable and intractable problems: problems that we can see how to make progress on, and problems that we can’t. Consciousness as a mark of the mental relates to the issues in the latter category. (A similar, but more encompassing, is the distinction between “problems” and “mysteries” introduced by Chomsky (1975, 1980, 1988, 2000)).

The functionalist theory of mind can again be seen to lie at the heart of the matter. It is not difficult to see the usefulness of consciousness, in other words: it is not difficult to ascribe a causal role to consciousness. Being able to make conscious perceptions, and being able to make conscious decisions based on the received information are traits that have obvious selective advantage. Having a consciousness, and being conscious, obviously matters. However, it is consciousness in the sense of awareness that is the focus here. Being aware of your surroundings enables you to coordinate your actions with the environment; being aware of yourself, your body and your mental states enables you to control yourself and your actions. Perception, memory, learning, emotions, decision-making, action control – all of these are important psychological phenomena under vehement empirical study. Conducting such research is not in any way straightforward, of course, but the phenomena are empirically tractable – that is why it makes sense to do such a research. And the reason for that is that you can easily see what sort of function all of these different kinds of states of awareness ultimately serve. Specifically, you can fairly easily see what would happen if certain psychological faculties would be added or removed, or enhanced or impaired; in other words: they make a causal difference to those endowed by them.

So, issues related to awareness and cognitive processing are deemed “easy” in the sense of being empirically tractable. But there are other issues related to consciousness that are supposed to be fundamentally different, and to be empirically intractable, and therefore to be particularly “hard.” In fact, the situation here bears a striking semblance to the problem of intentional content: you can ascribe functional role to intentionality, and you can ascribe a functional role to consciousness, but as soon as you do that, the things that you were originally aiming to explain, and the things that are characteristically mental, slip through your fingers and you end up staring at a mystery. What slips through your fingers in this case, is the subjective qualitative content of consciousness.

Let us now dub the aspect of consciousness that is left out by functional analysis *phenomenal consciousness*, and the aspect that is attainable by such an analysis *access consciousness* (cf. Block, 1995, 2007; also Dretske, 2004, 2007): consciousness in the sense of awareness makes both our surroundings and our own minds accessible to us. It is phenomenal consciousness specifically that is singled out here as a mark of the mental. So what then are the exact features of phenomenal consciousness that make it intractable to functional, and, therefore, to physical analysis?

There are two features in particular that sit ill with the wholly physicalistic understanding of the mind. These are the qualitativity and subjectivity of conscious mental states. You can make even more fine grained distinctions (cf. e.g., Van Gulick, 2016), but it can be maintained that any more fine grained analysis will ultimately reveal only sub-types of either qualitativity or subjectivity. Since the aim here is not to analyze consciousness, but to provide an analysis of those features of consciousness that set it apart from the physical realm as a distinctly mental phenomenon, this coarse-grained level of analysis is justified.

Again, like in the case of intentionality, the philosophical literature is full of vivid examples and thought experiments that are meant to demonstrate the puzzling nature of phenomenal consciousness. Perhaps the most cited and discussed of these is the “zombie argument” (Kirk and Squires, 1974; Chalmers, 1996, 2009, 2010; Leuenberger, 2008): it seems possible to us, or at least conceivable, that there could be creatures exactly like us, except for lacking phenomenal consciousness. In other words, it seems like the subjective qualitative features of our experiences – how it feels and looks to us subjectively – do not make a difference to behavior. Of course the fact that we are aware of things through our experiences – that our experiences give us access to information about our surroundings and ourselves – makes an immense difference, as already stressed, but the way that things are phenomenally presented to us in our awareness does seem functionally superfluous. The zombie argument leads one to approach this issue from the point of view of removing the phenomenal consciousness – the zombies are lacking something that we have – but you might as well ask: what would it take to make zombies phenomenally conscious? Suppose you managed to create a highly sophisticated version of Frankenstein’s Monster, or an android that would behave just like the rest of us. What is the extra ingredient you would need to add to make these entities capable of being in phenomenally conscious states? And moreover, how would you test whether they are in such states or not? It seems that when you start to make progress in answering such questions, you immediately fall back to addressing questions concerning consciousness in the sense of awareness.

Another often referred to thought experiment is qualia inversion (Locke, 1689; Shoemaker, 1975, 1982; Block, 1990): it seems, at least *prima facie*, that if you switch the qualitative content of your experiences, and replace them symmetrically with each other, you will have different qualitative experiences of things, but you will not exhibit any changes in your overt behavior. You would now experience red as green and green as red, for example, and your phenomenal consciousness would be dramatically changed, but as long as this inversion is systematic, it should not affect your behavior (cf. however, Dennett, 1988, 1991, 1993, 1994; Hilbert and Kalderon, 2000). Again, you can also take an epistemic perspective on this issue: how do you know that the qualitative contents of other people’s experiences are not systematically inverted? How would you test that?

The relevance of the epistemic perspective to this issue is particularly well demonstrated by the so-called “knowledge argument” (Jackson, 1982, 1986). Imagine a scientist, Mary, who specializes in color vision. Mary is not only brilliant, but extremely hard-working: she has educated herself of all there is to know about color vision, its psychology, biology, and physics. However, she has done this, and lived all her life, in a completely achromatic environment, and has therefore never had an experience with a chromatic qualitative content. Suppose that she gets a chance to change her environment and acquire experiences with such content, and suppose that she indeed changes her environment – has she now acquired some new information regarding color vision? On the one hand knowledge of the science of color vision remains intact; she already knew everything there was to know about it. On the other hand she now seems to be capable of having experiences that are dramatically different to what she was capable of having before. In other words, *objectively* everything is as before, but *subjectively* things have changed dramatically.

The hallmarks of the physical are objectivity and quantitativity. The objects of natural science are quantitatively measurable and open to objective scrutiny; the results of research are exact and publicly reportable. It seems that phenomenal consciousness is in direct conflict with both of these marks of the physical. Phenomenal consciousness is what it is *like*, and what it is like *for me*, to have an experience, and that’s all it is (cf. also Nagel, 1974). (It is worth noting that sometimes the subjective character of our mental states is singled out as its own epistemic mark of the mental (e.g., Rorty, 1970, 1979; Kim, 1971, 2011; Levison, 1983; Farkas, 2008a; Tartaglia, 2008). However, one can assume that such direct, first-person privileged access applies solely to states of phenomenal consciousness.)

The philosophical examples and thought experiments concerning phenomenal consciousness can easily lead one to think that there is a principled impossibility in explaining consciousness in physical terms – or indeed at all. However, that is not the intention here. The only thing that has been established is that phenomenal consciousness, its essentially qualitative and subjective nature, seems anomalous from the current physicalistic perspective; that there is an “explanatory gap” (Levine, 1983) in that our current physical explanations do not seem to reach the phenomenal consciousness. Some have indeed claimed that this gap can never be closed, and consciousness – the mental realm in general, but the phenomenal consciousness in particular – will remain as a mystery (e.g., Huxley, 1869; Chomsky, 1975, 2000; McGinn, 1989a, 1991, 1995; Pinker, 1997). But some are more optimistic, and think that both empirical and conceptual advances will ultimately solve the problem of phenomenal consciousness (e.g., Crick and Koch, 1990, 2003; Laughlin et al., 1990; Flanagan, 1991, 1992, 1998; Varela, 1996; Koch, 2004; Lamme, 2006, 2010; Block, 2007; Fahrenfort and Lamme, 2012). Others have an equally strong faith in natural sciences, but hold that rather than solving the problem, their advances will end up dissolving it – showing that there is really no such thing as phenomenal consciousness, or a special hard problem of consciousness, separate from the various faculties of awareness and the easy problems associated with it (e.g., Dennett, 1988, 1991, 1992; Dennett and Kinsbourne, 1992; Carruthers, 2000; Cohen and Dennett, 2011; Seth, 2016). Lastly, and perhaps most interestingly, some hold that the arguments show how consciousness is in fact an ubiquitous, primitive element of reality – that some sort of dualism or panpsychism is the right view to adopt (e.g., Nagel, 1979, 2012; Robinson, 1982; Foster, 1989, 1996; Seager, 1995, 2006; Chalmers, 1996; Rosenberg, 2004; Strawson, 2006; Tononi, 2008; Koch, 2012; Goff, 2017).

The reason why the panpsychic reaction to the hard problem of consciousness, or at least some formulations of the reaction, is particularly interesting is that this is one rare occasion where the tension between the mental and the physical is discharging at the cost of the physical. As already noted, if we take the juxtaposition between the two realms at face value, there are no obstacles for thinking it is in fact the physical that is particularly anomalous, rather than the other way around. The reason why the mental is typically bearing the burden of being anomalous is simply because we hold the physical perspective as primitive, sometimes explicitly, but more often implicitly. The panpsychic solution to the hard problem appeals to many because, first, the subjective qualitative features of consciousness seem so tangible and real that it is impossible to see – by those who are attracted by this view – how they could be eliminated or explained away, and second, the gap between the two realms seems so insurmountable that any attempts to give physicalistic explanations for phenomenal consciousness are bound to turn out to feel insufficient, or, maybe more appropriately: fundamentally off-mark. That is why the physical must give in, according to this view, and accept the mental to its side as a fundamental, primitive element of reality.

All these reactions to the problems posed by phenomenal consciousness are consistent with the view advocated here, namely that phenomenal consciousness is a paradigmatic mark of the mental. The panpsychic reaction fits in this particularly well because it holds on to the irreducible and ineliminable, and inexplicable, nature of the phenomenal consciousness so tightly. However, even the downright eliminativist views take the challenge of the hard problem seriously in the sense that they go through great efforts to explain the phenomenal consciousness away. So whether you are interested in defending the existence of the mental, or explaining it, or reducing or eliminating it, you will need to treat phenomenal consciousness as a mark of the mental.

Although one could thus find it appealing to single consciousness out as a special, prominent mark of the mental, it should still be stressed, however, that the issues of intentionality and consciousness are very intricately intertwined. There are at least four different positions one can hold, each with their own nuanced subsects. Many – the “intentionalists,” or “representationalists” – think that there is no separate, intrinsic, non-representational content to our conscious experience; that all our mental states are in fact representational (either directly or derivatively), and that once we have got the story about representations right, we have actually managed to solve all the problems we associate with the mental (e.g., Anscombe, 1965; Lycan, 1987, 1996; Harman, 1990; McDowell, 1994; Dretske, 1995; Tye, 1995, 2000; Carruthers, 2000; Kriegel, 2011). Some – the “anti-intentionalists,” “separatists” (Horgan and Tienson, 2002) or “phenomenists” (Block, 2003) – maintain that there is an independent, intrinsic and irreducible phenomenal aspect to our conscious experiences, and that hence both intentionality and phenomenal consciousness must be recognized as full marks of the mental (e.g., Peacocke, 1983; Block, 1995, 1996, 2003; Kim, 2005, 2011). Yet others stress the fundamental interdependence of intentionality and phenomenal consciousness; that separating the two, and the ensuing issue of whether they are independent, or which of them should be seen as more primitive or real, is misguided (e.g., McGinn, 1989b; Shoemaker, 1996; Siewert, 1998). And finally, there are those who see phenomenal consciousness as primary, grounding the representational content of our experience (Horgan and Tienson, 2002; Pitt, 2004; Farkas, 2008b; Pautz, 2013). It is often far from clear where the battle lines in this debate are drawn, and it is easy to emphasize different arguments and slide between the positions. However, the fact that these debates are raging, and they attract wide attention, is yet one indication of the importance of phenomenal consciousness as a mark of the mental.

## Free Will

Intentionality and phenomenal consciousness are the two typically recognized marks of the mental. Rarely are any other marks brought to their side, and rarely, if ever, is the issue of free will discussed in this context. This is very strange, for a number of reasons. First, freedom of the will is something very tangibly and intuitively mental; it is no doubt something that a layperson would readily offer as an essential feature of our minds. Second, numerous empirical and semi-empirical studies addressing the fundamental issues in the philosophy of mind are actually addressing, or at least claim to be addressing, the issue of free will. Third, free will creates a rather obvious juxtaposition between the mental and the physical: not many doubt that we have free will – not at least without a long litany of specifications and provisos – but few are ready to grant such powers to bare physical objects.

Now, it is more important than ever to choose one’s words carefully. It is by no means clear what the notion of “free will” is supposed to mean, and what the problem, and the proposed solutions, are exactly amounting to. There is thus a great deal of conceptual thicket to clear, even more than in the previous cases. One must also be careful not to confuse the analysis of the notion of free will with the project of defending its existence, or a particular interpretation of it. It is one thing to get clear what we should mean by the notion, be that one or many things, and another thing to show that it has a scope. As before, it might very well turn out that adopting some sort of an eliminativist view is the right way to go.

Traditionally, the main problem is the apparent conflict between free will and determinism: if everything that happens is completely determined by prior events, so too our decisions and actions, whatever we do, seem to be fully determined by prior events. For free will to exist, determinism would need to be false. But now problems arise. The falsity of determinism implies indeterminism – the two are supposed to be complementary opposites to each other, at least on typical interpretations. So free will appears to require indeterminism. But, as it is well-known, free will seems to be incompatible also with indeterminism: if the occurrences of future events are indeterminate, then they are not determined by our decisions; arbitrary events are not willed. So free will seems to require both determinism and indeterminism, but also to be in conflict with both of them.

There is no consensus on how to move forward. Incompatibilists stress the conflict between determinism, and seek – if they still believe that there is, or should be, such a thing as free will – to find ways to make free will compatible with indeterminism (e.g., Campbell, 1951; Chisholm, 1964; Lehrer, 1968; van Inwagen, 1983, 2000, 2004, 2008; Kane, 1999). Incompatibilists are thus typically incompatibilists with respect to determinism, but compatibilists with respect to indeterminism. Compatibilists, on the other hand, are eager to stress the conflict between indeterminism, and seek to find ways to make free will compatible with determinism (e.g., Moore, 1903; Schlick, 1930; Ayer, 1954; Smart, 1961; Lewis, 1981; Smith, 1997, 2003; Vihvelin, 2000, 2004, 2011, 2013; Berofsky, 2002; Fara, 2008). Compatibilists are thus typically compatibilists with respect to determinism, but incompatibilists with respect to indeterminism.

This is not a treatise on free will, and there is no point in recounting all the nuanced debates between compatibilists and incompatibilists. However, it is important to point to a central issue that connects the two camps. Both compatibilists and incompatibilists take moral responsibility as a non-negotiable starting point, and both tend to agree that moral responsibility requires free will, but depart on the issue of whether moral responsibility is in conflict with determinism. Incompatibilists think that in making decisions you must be facing genuine alternatives in order for you to be able to choose your actions freely and be responsible for your actions. Compatibilists think that no such genuine alternatives are required for moral responsibility. All that is needed is that you are free from coercion and that your actions are based the right way on your mental states and on your reasons for acting – that you have genuine moral agency. As determinism can be made compatible with all this, it is no real threat to free will. All this has some important consequences for assessing the role of free will as a mark of the mental.

The first thing to note is that although logic dictates that determinism and indeterminism should be on a par with each other – since they appear to be mutually exclusive alternatives and both in conflict with free will – it is the former of the two that occupies a special place in the debate. After all, it is their relationship with determinism that identifies the two camps: compatibilism is determinism-compatibilism and incompatibilism is determinism-incompatibilism. Determinism has thus a special role to play in defining the nature of free will, at least psychologically.

The conflict between determinism and free will holds also a key to understanding how free will can be singled out as a mark of the mental. Determinism is closely related to the notion of “causal determinism,” the idea that every event, or at least every event that has a cause, has a complete, sufficient cause – that the occurrence of the cause-event was all that was needed for the effect-event to occur, and that citing the cause would thus provide a full explanation of the effect. One of the core theses of physicalism, on the other hand, is the idea that the physical realm is causally complete: that each physical event that has a cause has a sufficient physical cause. All the physical events would thus seem to be completely physically determined. And if all our mental states and all our actions are necessarily physically based, then it seems that whatever we think, and whatever we do, is fully determined by the physical courses of events. Free will appears now as an anomaly because it seems to require that we would be able to break the physical course of events and inject a distinct, mental causal influence on the world.

There are two separate problems here, that get easily conflated. The first is the problem of mental causation: the question of how can the mental, *qua* mental, have an independent effect on the material world. The proviso “*qua* mental” is essential here. Perhaps nothing seems more concretely real to us than the ability to change things and affect the courses of events around us by simply acting according to our conscious decisions. That is, we have a very strong intuitive feeling that these actions are emanating from us as conscious, and self-conscious, subjects, and not as physical objects. Even if you would be convinced that the mental and the physical are in fact identical, this feeling cannot be easily erased. That is why free will, in the sense of autonomous mental causation, deserves to be singled out as a mark of the mental.

It is all but clear, however, how exactly the issue of mental causation and free will are related (cf. Bernstein and Wilson, 2016). One could be eager to think that mental causation, in the sense just described, is a necessary requirement for moral agency and free will (cf. e.g., Kim, 2007). However, at least some compatibilist accounts, which interpret free will simply as an absence of coercion, could leave room for free will without mental causation. That does not mean that the issue of mental causation isn’t relevant in its own right, of course – it would just be wrong to discuss it under the title of “free will.”

Note also that the problem of mental causation does not arise from the assumption of determinism, not at least without some further specifications. Rather, the root of the problem is in the assumption that the physical level is causally complete (and that mental states are fully based on physical states). Supposing that mental causes are not systematic overdeterminers – that in each purported case of mental causation there exists both a full physical and a full mental cause for the given effect – mental causes seem to be left with a wholly otiose role, and they would thus become excluded by physical causes (cf. Kim, 1989, 1998, 2005). Nothing in this result seems to hinge on determinism, not *prima facie* at least, but only on the causal completeness of the physical. (However, as already noted, if causal completeness is cashed out in terms of causal sufficiency – all physical effects having sufficient physical causes – and if that is then interpreted to mean that nothing else but the occurrence of the cause-event accounts for the occurrence of the effect-event, one comes awfully close to saying that given the occurrence of the cause-event, the effect-event was determined to occur. Much thus depends on how the notion of causal sufficiency is to be understood in this context (cf. Suppes, 1970; Anscombe, 1971; Pernu, 2013)).

If it is the causal completeness of the physical that is at the heart of the matter here, it is possible to formulate the tension between the mental and the physical in a particularly precise way. Although it would be too much to say that the two principles are identical, the idea of the causal completeness of the physical has apparent affinity with the first law of thermodynamics, the idea that the total energy of a closed system remains constant (cf. Papineau, 2002). Given that all physical events are either identical with or based on energetic changes, all physical events would now always correspond to other quantitatively equal physical events. Whatever mental states these physical events would now base, they would be prone to appear causally superfluous. It is not really relevant whether this train of thought can be made completely watertight – one could point out, for example, that the causal completeness of the physical is a metaphysical thesis about causation and causal explanation, whereas the principle of the conservation of energy is a physical principle based on mathematical symmetries; there are obviously gaps to fill. However, this does not have to erode the intuitive connection between the two principles which explains, at least for a large part, our difficulties in comprehending the idea of autonomous mental causation.

However, mental causation is only part of the larger problem the notion of free will is imposing on us. The problem of mental causation is the problem of how the mind can, *qua* being mental, have an effect on the future events of the world – or, to formulate the issue in more poignant terms, the problem of how the will, *qua* will, can have such powers. But of course the problem of free will is more than the problem of the will: it is, first and foremost, the problem of the *freedom* of the will. And it is here where the conflict between free will and determinism becomes most apparent: how can your choices, your will, be yours, and how can you be free to make those choices, and will what you will, if everything is determined by things and events out of the scope of your influence? This is the core question of free will, and the one that motivates the debates surrounding the incompatibilist approaches to the issue. So even if you would be able to offer a convincing solution to the problem of mental causation, many would insist that you would still have left the main issue completely untouched.

If the freedom of the will is thus singled out as a source of a tension between the mental and the physical, it becomes pertinent to ask if the threat of determinism is merely hypothetical. After all, according to the widely accepted paradigm the fundamental physical reality is in fact indeterministic. Should we thus ignore the threat of determinism, and maybe start building our view on indeterministic quantum physics, as many are eager to suggest (e.g., Eccles, 1994; Penrose, 1994; Hameroff and Penrose, 1996; Beck and Eccles, 1998; Stapp, 1999, 2009; Schwartz et al., 2005)? There are many reasons to be skeptical of such a project (cf. Pernu, 2011). First, simply trading determinism for indeterminism would not get us far: we would still need to explain how our conscious decisions arise from the random quantum events. Secondly, and more importantly, the physical level that is relevant to this explanatory project is fully accountable by classical terms. This is not just an issue of all the physical processes relevant to mental functioning taking place at such a coarse level that all the quantum indeterminacies will be canceled out (Tegmark, 2000). The main critical point is that turning to quantum physics overshoots: “the physical,” that is in apparent tension with “the mental,” is referring to a much broader category than to mere fundamental physics. “Physical” and “physics” are distinct notions, at least in this context, with the former referring to a variety of macrophysical entities, such as tissue, organs, and bodies. Whatever conscious decisions are to be correlated with, they are bound to be some features of neural networks, not single neurons, let alone their microphysical parts.

To complicate things even more, it seems clear that in addition to the problems of mental causation (the problem of the will) and the problem of the freedom of the will (the problem of freedom), free will is also related to the problem of consciousness; intuitively, conscious decision making is a necessary condition of free will (Shepherd, 2012, 2015). However, it should be fairly incontestable that it is not consciousness in the sense of phenomenal consciousness, but in the sense of access consciousness, that we are facing here: we need to be, at least *prima facie*, aware of our decisions and actions in order for us to be acting on the basis of free will. There are at least two separate issues here. First, it seems that free will requires a sense of selfhood, that the subject of free will is conscious of herself as an autonomous agent and a source of her actions. Second, it seems that the subject needs to be aware of her decisions and the actions she is making based on her decisions – she needs to be acting purposefully, with an intentional effort to produce specific outcomes. In other words, free will seems to require both *self-awareness* and *action-awareness* (Gallagher (2000a,b, 2008, 2015) proposes a related distinction). These two aspects of awareness account for the feeling that our actions, at least the ones that we deem free, are “up-to-us”; that there is a proper sense of autonomous control associated with the actions that are the results of our free choices.

A number of empirical studies, conducted in various different ways, have recently proposed a startling conclusion: that our actions do not in fact result from our free choices, but that it merely appears to us so – that we are under the spell of “willusionism” (Libet, 1985, 1994, 2002, 2003, 2004, 2006; Wegner, 2002; Prinz, 2003; Lau et al., 2004; Soon et al., 2008; Harris, 2012). Such a conclusion threatens to leave us zombies yet in another way: now it seems that the thoroughly physical (neural) description of our behavior leaves us without conscious control over our actions (Vierkant et al., 2013; Shepherd, 2016) (see **Box 2**). Whether these arguments are correct – and there are many cogent ways of challenging them (e.g., Nahmias, 2002, 2011; Pereboom and Caruso, 2002; Levy, 2005; Waller, 2012; Clark et al., 2013; Mele, 2014a,b) – it is worth making clear that what they are really targeting is our conscious sense of free will, rather than the idea of free will itself. That is, these arguments might, if correct, pose serious challenges to the idea of the causal efficacy of the will, or at least to the assumption that the actions we deem free bear a necessary connection to conscious decision making. So although these argument might not pose a direct challenge to the idea of the freedom of the will, they do create a real ground for concern since it seems rather obvious to us that conscious decision making is a necessary element of free will.

**Box 2. Physicalism and three kinds of zombies.**

A thoroughly physicalistic view of ourselves threatens to make us zombies in at least three distinct senses.

**Semantic Zombies.** The physical world seems to be governed by wholly syntactic, mechanical processes, leaving the semantic features of our mental states – the content of our desires, beliefs, and perceptions – without any causal role, and transforming us thus into syntactically driven zombies.

**Phenomenal Zombies.** Similarly, the subjective qualitative contents of our conscious mental states, the way that things feel and seem to us in our private experiences, seem to be left causally inert from an objective, physicalistic point of view deployed by the sciences, thus prompting us to treat ourselves as phenomenal zombies.

**Free Will Zombies**. Finally, recent empirical studies on free will have suggested that our conscious decisions do not have a role to play in the initiation of our actions, stripping us of conscious control over our behavior, and making us thus neurobiological zombies, devoid of any true agency and free will.

Things are not that straightforward, however. Although many accounts of free will (and consciousness) take it for granted that consciousness is a necessary requirement of free will – to the point where many treatments of consciousness are not actually addressing the issue of consciousness at all, but the issue of free will (and vice versa) – a moments reflection shows that the connection isn’t necessarily as tight as it seems. As it was noted, it is typically agreed that the idea of free will is strongly connected to moral responsibility, so strongly that many are prepared to make free will compatible with determinism in order to save moral responsibility. And, as it was also already noted, this would also seem to allow the possibility of acting freely even in the absence of mental causation. And if that is the case, then it seems clear that it is also possible to act freely in the absence of consciousness. This is actually not so preposterous as it might first seem. For example, we often hold people accountable for actions committed under the influence of intoxicants. In general, loss of memory, or otherwise dramatically impaired cognition does not necessarily make us strip the subject of free will. Or consider absent-mindedness, or routine actions. We do various different things absent-mindedly: we dress up, have a walk, do shopping, and even conduct conversations without being fully aware of what we are doing. Yet, we assume that all these things are done perfectly freely – we are, after all, blameworthy for our absent-mindedness. Or consider the case of playing an instrument. When you are practizing a song to play, you need to concentrate on your playing and on making very fine grained motor actions. However, after you have mastered the song, and you play it routinely, you are not aware of all these fine grained actions any more. Yet, it wouldn’t seem right to say that you are not acting freely when playing the song and acting that particular way. A credible case could thus be made for severing the necessary connection between free will and consciousness.

It can nevertheless be maintained that consciousness, both in the sense of self-awareness and in the sense of action-awareness, is a feature typically associated with free will, and one of the factors that adds to the feeling that the mental realm is strongly distinct from the physical realm. One could even hold on to the idea of a necessary connection between free will and consciousness by devising an argument showing how in cases where the connection is putatively severed, you can actually always trace the chain of events to a point where a fully conscious decision was made (e.g., you consciously chose to consume the intoxicants, and you consciously chose to practice the song and perform it routinely – you didn’t do these things by mistake, and nobody forced you to do them).

One could thus conclude that there are three distinct components in the idea of free will that create the tension between the mental and the physical: awareness of the will, causal efficacy of the will, and freedom of the will. It seems fairly clear that the first one of these reduces to a special case of access consciousness, to self-awareness and action-awareness. The latter two, however, are more at the core of the identity of free will as a distinct mark of the mental (cf. Watson, 1987; Ekstrom, 2011). The causal efficacy of the will is a problem due to the physical realm being causally complete and thus excluding any distinct mental influences. The freedom of the will is a problem due to the physical realm being governed by deterministic laws, thus leaving no room for the conscious will to choose among alternative courses of actions.

## Teleology

The notion of teleology is also something that does not figure in typical discussions on the marks of the mental. And, again, this is rather strange. Folk psychology routinely employs teleological notions in explaining our behavior: we act in the way we do because we have particular goals in mind. But purely physical explanations eschew such teleology: the occurrence of events are explained by citing antecedently occurred events, not by citing the events that occurred consequently. Natural processes are not goal-directed, conscious behaving essentially is. There seems to be quite an obvious tension between the mental and the physical views on the world.

Teleology has rather apparent links to the previously introduced marks of the mental. First, many intentional states seem to have a teleological component in them: your thoughts are directed to the future events – you believe it is going to rain later, and you desire not to get wet then – which explains your current behavior – that you took an umbrella with you. Most blatantly: most of our actions are intentional – we strive for the results of our actions purposely. Second, “acting intentionally” seems to be almost equivalent to “acting consciously”: when we act purposely we act with certain goals in our minds – in other words, we are aware both of the goals and our strive toward them. Third, “acting intentionally” seems also to be almost equivalent to “acting in accordance with free will”: not only are intentional actions accompanied by the sense of awareness that they originate from conscious decisions, they are also morally aggravating. Teleological notions are thus at the heart of mentality.

It is instructive to approach this theme from a historical point of view. One conspicuous feature of the evolution of the natural sciences is the gradual decline of teleological notions. Aristotle, most famously, took teleology to be the corner stone of scientific explanation (e.g., Johnson, 2005; Leunissen, 2010). Entities have, according to him, natural tendencies of being in change or being in rest. The effect of gravity on material objects, for example, is explained by pointing to the natural tendency of objects to move toward their natural places, i.e., toward the surface of the earth. Moreover, the full explanation of events, at least in most typical cases, must cite all the four distinct causes of the events: efficient, final, formal, and material. Of these the second, final cause, is explanatorily prior: although citing all these causes amount to different ways of replying to the question “why?,” it is replying to the question in the sense of “what for?” that is most revealing to us. The main reason for this is that answering such questions will give us understanding of the regularities observed in nature – such as material objects always falling to the ground.

It is important to realize that appealing to teleological notions is perfectly natural for Aristotle. In other words, there are teleological, natural tendencies present in nature and in objects themselves; teleology is not a psychological phenomenon to him – although appealing to final causes is particularly revealing in psychological contexts – and he is not claiming that there is some sort of extraphysical conscious guidance present in nature. Teleology in nature is neither borrowed from our psyche nor imposed by something supranatural, but natural processes are inherently teleological. One could thus claim that Aristotelian naturalism is the first attempt to get a grip on the apparent teleological phenomena surrounding us in wholly naturalistic terms.

The rise of mechanical physics abolished teleological notions from physics, but they seemed to be harder to root out from many other fields of science. Biology in particular was, and still is, rife with teleology. In fact, one could raise the question whether it would be more appropriate to identify teleology as a mark of the living rather than as a mark of the mental. There are at least three ways in which we become faced with teleological notions in biology. First, the behavior of organisms seems teleological: they seek to find nutrients and mates, and to avoid hazards and predators – organisms strive to survive and reproduce. Second, the ontogeny of organisms seems teleological: most complex organisms develop from very simple ingredients, and very reliably so. Third, and most importantly, the functioning of organisms and the whole evolution of nature seems teleological: organisms have traits that enable them to function in appropriate ways, species match their niches, and there seems to be a natural hierarchy from simple organisms to more complex ones – with us humans at the top. In other words: nature seems designed, perfectly tuned, in various different ways.

The main lesson of Darwinism, of course, is that all this biological teleology surrounding us should be understood as merely apparent. What Darwin (1859) showed is that we can make sense of nature in perfectly mechanical, causal terms: species and their traits are simply results of natural selection. A central concept of the Darwinian theory is adaptation: organisms behave in a seemingly teleological manner, and have traits that seem to serve a purpose, because they have been adapted to behave in these manners, and have such traits. More specifically: organisms behaving in these ways, or having these traits have survived and reproduced in higher rates than other organisms, and they have passed these ways of behaving, or these traits (or slight variations of them) to their offspring. All the biological diversity, and the seeming teleology in it, can be fully explained by a differential rate of reproduction, which in turn is perfectly in line with the mechanistic understanding of physics and chemistry.

Given that we have in this way stripped physics and biology of teleological notions, and that this development has been one of the crucial reasons – if not *the* crucial reason – for the success of modern science, it becomes pertinent to ask if the teleological notions present in psychology could, and should, also be given a critical treatment. This indeed has been happening. The key to this development has been the gradual progress that has been made in analyzing functions and functional explanation in causal-historical notions, akin to natural selection.

Although there are significant conceptual issues to tackle, and the philosophical work in this area is ongoing, a relatively clear progression can be outlined. First, the theory of evolution by natural selection gives a natural – causal-mechanical – explanation of biological functions (behavior, traits, and organs). Consider the paradigmatic example: the function of heart is to pump blood (rather than produce a thumping sound). The reason why it makes sense to ascribe functions like this to organs is that we can easily see how they have been evolved to have these functions due to them accruing fitness benefits to the ancestors of the organisms that now have these organs – the heart has been selected for pumping blood because having such organs enhanced the organisms likelihood to survive and reproduce.

The next step is to fit psychology and mental functions into this scheme. First, mental faculties can be seen as perfectly analogous to bodily organs: perception, memory, learning and other mental capacities have been selected for their beneficial functions – as already discussed, it is fairly easy to understand the ecological usefulness of access consciousness. Second, and maybe even more importantly, there are various attempts to solve the problem of representational mental content in these terms. Roughly, the idea in these teleofunctional or teleosemantic theories of mental content is that we can understand representational capacities through their selectional history: mental states have acquired via natural selection or learning the function to represent spatiotemporally displaced state of affairs (Millikan, 1984, 1993, 2000, 2004, 2005; Papineau, 1984, 1987, 1993; Dretske, 1988, 1995; Sterelny, 1990; Price, 2001; MacDonald and Papineau, 2006; Neander, 2017).

None of this has to do anything with adaptationism – the idea that all traits have a function for which they have been selected – or with evolutionary psychology – the idea that all psychological and social phenomena should be given an evolutionary explanation. These are controversial theses that are related to more specific concerns about where and how evolutionary explanations can, and should be applied. The focus here is rather on the completely general idea, that is practically uncontested, that appealing to selectional explanations can give us a perfectly natural understanding of how psychological phenomena can be fitted into a causal-mechanical picture of the world.

Although it is safe to say that there is thus a clear historical trend of renouncing or weakening the role of teleological notions in scientific explanation, it is not clear what exactly this development amounts to. One could claim that with respect to physics and basic natural sciences it would be correct to say that teleology has been eliminated (however, it is interesting to note that some remnants of teleological conceptualisation can still be interpreted to be present in some corners of physics, most notably in discussions concerning the anthropic principle and the second law of thermodynamics (e.g., Carter, 1974; Wicken, 1981; Barrow and Tipler, 1988)). However, when we turn to biology, it might be more correct to say that rather than eliminating teleology, the modern understanding of biology has explained, or maybe reduced it. Many debates are raging on how exactly to analyze the notions of natural selection, fitness, function and adaptation, and what their relation to the teleological interpretation of these notions is (cf. Cooper, forthcoming). Mayr (1974, 1988, 1992, 1998, 2004) in particular has vehemently defended the place of teleological notions in population biology; John B. S. Haldane has been know to have quipped that “teleology is like a mistress to a biologist: he cannot live without her but he’s unwilling to be seen with her in public” (in Mayr, 1988, p. 63).

When one moves from biology to psychology, it should become apparent that there is a rather obvious metaphysical connection between the two: both biology and psychology are thoroughly entangled with informational notions, and both are focusing attention on self-regulating processes and systems. Consequently, the field of cybernetics was established to study the interconnection – and interaction – of the two domains (e.g., Wiener, 1948, 1950; Crosson and Sayre, 1967; Sayre, 1976; Bynum and Moor, 2003). The exact meaning of “cybernetics” is notoriously elusive, of course, and the field is highly diverse. What is noteworthy, however, is that there was an explicit recognition of the need for a systems-level analysis of natural – and artificial – phenomena. This in turn made teleological notions, and the mind-body problem defined in terms of them, to creep up to the center of attention once again. On the one hand it could be claimed that the cybernetic explanation of self-regulating and self-controlling systems can be understood in perfectly physical terms: there is nothing mysterious about homeostatic behavior based on various feedback mechanisms. On the other hand one could insist that there is an irreducible informational element to such phenomena: “[i]nformation is information, not matter or energy. No materialism which does not admit this can survive at the present day” (Wiener, 1948, p. 155). But whether cybernetics is seen as an endeavor to explain or reduce teleology, or whether it is rather taken to show how teleology can, and must, be accommodated in our scientific world view, is not essential. Whichever side is interpreted to be the one that is doing the giving in, the cybernetic tradition is yet another proof of how the push from the side of teleology is concrete and strong.

So even if a strong case could be made for the elimination of teleology from psychology, one could say that the jury is still out, and the verdict might eventually be more favorable to teleology. Even downright realistic interpretations have been suggested (e.g., Schueler, 2003; Sehon, 2005, 2016; Goetz, 2008). However, as before, the main issue here is not the question whether teleology will become eliminated or reduced, or whether a realistic attitude is the right one to adopt, but the fact that the teleological way of explaining our behavior is very natural to us, and that it is in a stark contrast with the causal way of explaining physical phenomena. That is why it should be singled out as a mark of the mental.

## Normativity

Normativity is again something that does not appear in typical discussions of the mind-body problem, at least not directly (Zangwill (2005, 2010) is an important exception). On the one hand this is very understandable: the issue of normativity is complex, deeply intertwined with all the previously discussed issues, and hence not easy to give an independent characterisation. On the other hand this is very unfortunate: it could be argued that normativity is something that forms the core of many philosophical problems relating to psychological explanation, and it is also something that creates tension between the physical, or naturalistic, way of understanding the mental phenomena in a unique and particularly profound manner.

So what is normativity and how should it figure as a mark of the mental? Normativity relates to norms, to what is considered to be right or correct, and to what ought to be, in contrast to what merely happens to be. The general characterisation of normativity as a mark of the mental is this: there seem to be normative constraints, utilized in various different ways, on how to ascribe mental notions and attribute mental states to subjects, and such constraints are constitutive to the mental states. The tension that arises from this is, of course, that the purely physical view of the world is not supposed to contain such normative elements. Description and prescription are fundamentally distinct, and how things are has very little bearing on how things ought to be – let alone the other way around.

To see more clearly what’s at issue here, and to approach the problem systematically, let us address two different questions. First, what, exactly, creates the tension between the normative and the purely physical views on the world? And second, given that there is such a tension, what does it have to do with psychological explanation, and the tension between the mental and the physical? The tension between the normative and the physical stems actually from an even deeper tension between the normative and factual: from the apparent impossibility of deriving norms from purely factual premises. There seems to be a logical gap between these two: no matter how things are, it is always possible to ask further how they should or ought to be (Hume, 1738; Moore, 1903). Since this seems to be a purely conceptual or logical result, it does not have anything in particular to do with physicalism – idealism and dualism would be equally ill-suited metaphysical doctrines for deriving normative conclusions. Physical way of describing the world is just one factual way of describing the world, and the tension between the normative and the physical arises from its factual nature, not from some specific metaphysical theses connected to physicalism.

The connection between normativity and the mental is more complicated. One can begin by noting that normativity considerations are ubiquitous in human interactions. Language and language use is one particularly clear and concrete example of our normative practices, and one that is quite directly linked to psychological explanation. First of all, natural languages are essentially conventions, in syntax, semantics and pragmatics. Mastering a language is essentially an issue of mastering a rule, or a set of rules; there are right and wrong ways of forming expressions and using language. Secondly, there is a continuum in which people can be said to be able to speak and use a particular language. In other words, there is a set of criteria – a vaguely defined and tacitly utilized set of criteria of course – that we use to assess whether a person is able to use a particular language.

Now, one can quite confidently state that there is a tight and direct connection between language and the phenomena and processes we consider mental. Although there are profound debates on whether there is such a thing as a “language of thought” (Fodor, 1975, 1987, 2008) or whether “private language” is impossible (Wittgenstein, 1953; Kripke, 1982; Mulhall, 2007), it should be quite clear that many psychological phenomena have language-like characteristics, and it isn’t necessarily relevant whether such characteristics are internal to some language of thought or whether they are externally imposed on us. Not only does it seem natural for us to characterize other people’s psychological states in linguistic terms, it is also a way for ourselves to become aware of our own mental states and their roles in our thinking (cf. Davidson, 1974). But the way in which such linguistic considerations bear relevance to various mental phenomena is only one fairly obvious way in which normative considerations mesh with psychological explanation.

There are at least two distinct and more deep-cutting ways in which normativity penetrates psychology, and creates tension with a purely physical view of reality. Firstly, normative issues are closely linked with the issues related to teleological explanation. In fact, the reason why teleological notions are difficult to apply to purely natural contexts comes down to, at least partly, to the fact that they have a normative element to them. As discussed, it is tempting to analyze teleological notions in functional terms and then give functions an analysis in terms of natural selection (or some other causal-historical process). Organs, for example, do many things, but the things they are meant to do – the things they *ought* to do – are determined by their selectional history. Similarly, it can be argued that our mental faculties, and the semantic content of our mental states in particular, are determined by their selectional history. In other words, by appealing to natural selection – or to other historical and purely causal-mechanical chains of events – we can define perfectly natural criteria of the correct and incorrect application of psychological notions.

One reason why this type of reasoning is particularly relevant to psychological contexts is that it does not only give us a natural definition of function, it also gives us a natural definition of *dys*function or *mal*function – natural criteria of when we can correctly, and objectively, say that something has gone *wrong*. This opens a way to understand diseases in natural terms: diseases are deviations from the norm set by natural selection (Boorse, 1975, 1976, 1977, 1997). Having such an objective definition of disease would of course be tremendously helpful in defining the proper scope of medical interventions. Extending this approach to psychiatry and psychology would be particularly useful: we would finally have objective and operative definition of mental disorder and illness (Wakefield, 1992, 1997a,b).

It is easy to see, however, how this line of thinking is bound to face problems. Many medical conditions, mental disorders and illnesses in particular, have a strong social and cultural component to them. Is homosexuality a disease or a disorder? The problem is not just that answers to such questions seem to depend more on our values than on simple biological facts, but that nature itself always contains variation in all traits. In fact, natural variation – that there are differences in traits, at various scales, and at different levels of biological organization – is one of the necessary conditions of evolution by natural selection: there has to be variation in traits for there to be variation in fitness, which in turn can then lead to cumulative evolutionary change. Natural selection requires a variety of things to choose from. Although the theory of evolution by natural selection can give us understanding of how certain types of traits can become prevalent in a particular population at a particular time, it might seem quite of a leap to elevate this simple fact of biology to the role of a normative yardstick.

Again, it is worth reminding that the issue here is not whether normativity – in general or in the specific sense displayed in functional explanation – can ultimately be naturalized. Maybe such a project is feasible. However, that does not obviate the apparent resistance of normativity to naturalization. The focus here is simply on the fact that this resistance gives us a reason to treat normativity as a mark of the mental.

There is another, although a related way in which normative considerations enter into psychological explanation and come into tension with the physical view of the world. This is the much discussed dichotomy between causes and reasons (Davidson, 1963; Setiya, 2011; D’Oro and Sandis, 2013), or with explanation and understanding (von Wright, 1971). In explaining human behavior we are typically appealing to reasons a person is holding for behaving in a certain way; we understand, or make sense of actions by embedding them into a conceptual and socio-cultural scheme. Causal-mechanical explanation of the world lacks such elements.

Here is an example to demonstrate the stark contrast between causes and reasons in explaining behavior. I travel a lot, and I’m constantly flying to different places around the world. Often when I’m on a plane there are babies on board, and quite often they are crying, especially during the take-off and landing. Since this has been happening so often, I started to wonder if there is a reason for this behavior, and I decided to look this up. And it turns out that there is indeed a reason for this: the babies are crying because they are upset from gay-people getting married (cf. Székely, 2015). Now, why does this sound absurd? Mainly because we know that babies can’t have such reasons for their behavior, in fact, we don’t think that babies act according to reasons at all. We also know that there is a perfectly good causal-mechanical explanation for this type of behavior: the sudden changes in air pressure causes an uncomfortable and sometimes painful feeling in your ears. You can try to alleviate this only by actively leveling the pressure. Infants don’t know this, of course, and there is no way of explaining this to them. They cry simply because they feel *physically* uncomfortable. But adults often cry because they feel *mentally* uncomfortable; they have reasons for being upset, and we can try to understand and confront those reasons – and we can question and criticize them.

This cuts into the very heart of the dualistic, dichotomous image of ourselves as both physical and mental entities. We can have both causes and reasons for crying, and in the latter case we move into the social and cultural realm to explain the behavior. And this realm in turn, and in fact the very distinction between causes and reasons, is thoroughly normative. Whether it makes sense to cry, for example, does not only depend on some privately held reasons for behaving that way, but on the whole socio-cultural context in which the behavior occurs. In other words, there are rationality constraints to our behavior: whether we are seen as psychological agents – and to what extent we are seen as such agents – depends on whether our behavior can be seen to meet some norms of rationality. This is the reason, or at least one of the main reasons, why we are often reluctant to view animals as psychological agents: their behavior is too far removed from the norms we have set for psychological agency. Or, to make the same point other way around: pet animals that have co-evolved with us – cats and dogs – are sometimes eagerly granted such agency exactly because they respond to our psychological and social cues in a systematic and reliable manner, or so at least it seems to us. But more importantly, similar normative considerations are at play when we assess each other’s mental states. If we find it difficult to put the things that a person says and does into a scheme of reasons, we are prone to strip the person of her psychological agency – we can actually say that the person has “lost her mind.” Indeed, irrationality is the hallmark of mental disorders and illnesses, and rationality considerations bear heavily on our assessments of moral responsibility. Norms of rationality play thus a key role in us ascribing mental features to the surrounding world.

Further evidence for the thesis that normativity is at the center of the demarcation between the mental and the physical can be gathered by pointing to the links that this issue has to the previously discussed marks of the mental. Teleofunctionalism, or in general views that seek to explain mental content in terms of selectional histories, are the strongest contenders for solving the problem of intentionality. What really stands in the way, it seems, is the issue of whether mental functions can be analyzed in purely natural terms, eschewing normativity. Normative considerations are also clearly linked to the issue of access consciousness: whether, or to what extent, we are ready to ascribe such states to subjects – whether we are ready to say that subjects are aware of their surroundings and their own mental states – depends, at least partly, on how well the subjects’ behavior can be seen to accord with some preset norms of rationality, or “norms of awareness.” And similarly, there is an apparent connection between norms of rationality and free will: rationality considerations seem to bear on all the different aspects of the free will issue, on ascribing self-awareness and action-awareness, and on both whether subjects can be said to act according to their will and on whether they can be said to act freely. Rationality considerations bear on psychological agency, which in turn bears on whether, or to what extent, we ascribe free will and moral responsibility to subjects. Lastly, the teleological element in psychological explanation can, according to many, be understood in functional terms, which in turn leads one again by the question of whether functions can be analyzed without relying on normative notions. There is also an apparent connection between reasons and purposes: we act according to reasons, and those reasons only make sense in the light of the conscious purposes we hold. Teleology is closely associated with rationality, and one of the main sources of our reluctance to accept teleological notions in purely naturalistic contexts is based on our reluctance to impose objective rationality on nature, and to take natural processes to be governed by reasons rather than by causes.

There is an interesting outlier though: ascribing states of phenomenal consciousness does not seem to be based on normative considerations (however, cf. Kriegel, 2010). There is a fairly obvious reason for this, and the very same reason that singles phenomenal consciousness out as a mark of the mental, namely its apparent resistance to functionalisation. One could thus conjecture that the normativity considerations at the core of the other marks of the mental are largely related to functional explanation, and even if we would become content with a perfectly naturalistic analysis of functions, phenomenal consciousness would still be left out as an unanalysed, irreducible mark of the mental (*pace* Kim, 2005).

## Conclusion

**FIGURE 1:**
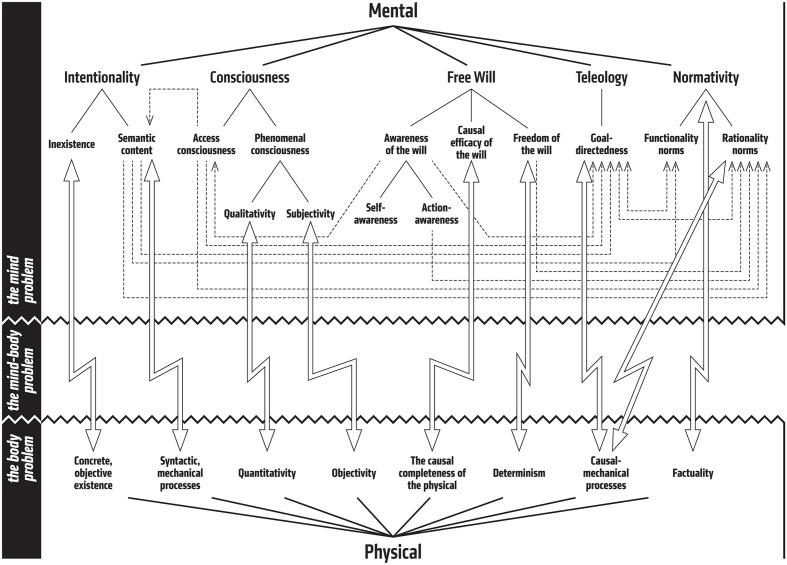
A figure summarizing the various components of the mind-body problem. The vertical lightning arrows represent the different points of tension between the paradigmatically mental and physical features. The dashed arrows within the mental realm outline some of the various interdependencies between the separate marks of the mental (the direction of the arrow represents the direction of dependence).

There is no such thing as “the” mind-body problem. There is a set of interconnected issues that together conspire against the purely physical understanding of the world (see **Figure 1**). One result of this is that the different components of the mind-body problem could end up solved, or dissolved, in different ways. Some marks of the mental might become reductively explained, others might become eliminated, and yet others might retain their identity as characteristically mental features and become realistically interpreted (see **Box 3**).

**Box 3. A break down of the different attitudes to the mind-body problem.**

At least the following five different ways of reacting to the mind-body problem can be distinguished:

**Dualism (realism).** The mental and physical realms of reality both exist and are equally real; the mental is distinct from, and not dependent on, the physical.

**Identity theory.** The mental and the physical are identical; there is a semantic distinction to be made, but the two are ultimately the same. Identity is a symmetric relation, so not only is the mental identical with the physical, but also the physical is identical with the mental.

**Reductionism.** The mental is dependent on, and nothing distinct from the physical; all mental features can be given a complete explanation in physical terms. Reduction is an asymmetric relation, so only the mental reduces to the physical, but not the other way around.

**Eliminativism (antirealism).** Only the physical realm is ultimately real; there are no mental features and mental notions should be eliminated.

**Revisionism and co-evolution.** Mental notions will become radically revised; they will not be eliminated, but we will start using them differently and they will start referring to other things than before. There will be co-evolution between the physical and biological understanding of the mind on the one hand, and psychological and social understanding on the other, and there is no *a priori* way of saying which of our mental notions will face elimination and which will become reinterpreted.

Decisions between reduction, elimination and realism cannot be made *a priori*. However, there is one important general note to be made. It became apparent in many places that the marks of the mental are very resilient in nature, that is, they seem to be resisting elimination at the cost of the intuitive meaning and metaphysical role of the notions. In other words, we hold on to the notions, and rather make radical reinterpretations and conceptual bending than eliminate them. Free will gets to be interpreted in terms that are compatible with determinism, teleology in terms of selectional histories, and phenomenal consciousness in terms of panpsychism, and so on. Mental notions are simply so dear to us, and so intimately connected with our everyday practices, that they are bound to be explained, one way or another, rather than eliminated.

There is a lot at stake – nothing less than the image of ourselves as consciously feeling and acting, autonomous psychological agents. It is thus understandable that eliminativism will meet resistance. However, this does not have to make one to turn to dualism. Mental notions could rather follow the fate of vitalistic notions: we could come to see how there are no separate “mental entities” or “mental realm” or “mental powers,” like we have learnt that there are no separate vital spirits or forces. Such a view does not have to amount to entailing that there is no real science of psychology. After all, biology as a field of science is now stronger than ever – and exactly because we learnt to connect the biological realm to the physical realm. A lot of discussion in philosophy of psychology has gone awry in this respect. It is a mistake to think that for a science to be autonomous it needs to posit its own irreducible substances or forces. Interdisciplinary autonomy can be seen as a wholly epistemic issue: making observations and constructing theories at different levels of abstraction is simply useful to us. Psychology, in particular, gains us useful information at the level of high biological – and social – complexity. This should be enough to defend the role of psychology as an autonomous scientific discipline. But it is a whole other question, of course, whether this will be enough to dissolve all our metaphysical worries relating to the image of ourselves as autonomous psychological agents.

## Author Contributions

The author confirms being the sole contributor of this work and approved it for publication.

## Conflict of Interest Statement

The author declares that the research was conducted in the absence of any commercial or financial relationships that could be construed as a potential conflict of interest.

## References

[B1] Anscombe G. E. M. (1965). “The intentionality of sensation: a grammatical feature,” in *Analytic Philosophy*, ed. Butler R. J. (Oxford: Basil Blackwell).

[B2] Anscombe G. E. M. (1971). *Causality and Determination: An Inaugural Lecture*. Cambridge: Cambridge University Press.

[B3] Arnau E. Estany A. González del Solar R. Sturm T. (2014). The extended cognition thesis: its significance for the philosophy of (cognitive) science. *Philos. Psychol.* 27 1–18. 10.1080/09515089.2013.836081

[B4] Ayer A. J. (1954). “Freedom and necessity,” in *Philosophical Essays*, ed. Cahn S. M. (New York, NY: St Martin’s Press).

[B5] Balázs É (1964). *Chinese Civilization and Bureaucracy: Variations on a Theme*. (trans. Wright H. M. ed. Wright A. F.). New Haven, CT: Yale University Press.

[B6] Barrow J. D. Tipler F. J. (1988). *The Anthropic Cosmological Principle.* Oxford: Oxford University Press.

[B7] Beck F. Eccles J. C. (1998). Quantum processes in the brain: a scientific basis of consciousness. *Cogn. Stud.* 5 95–109.

[B8] Bernstein S. Wilson J. (2016). Free will and mental quausation. *J. Am. Philos. Assoc.* 2 310–331. 10.1017/apa.2016.7

[B9] Berofsky B. (2002). “Ifs, cans, and free will: the issues,” in *The Oxford Handbook of Free Will*, ed. Kane R. (Oxford: Oxford University Press).

[B10] Block N. (1990). Inverted earth. *Philos. Perspect.* 4 53–79. 10.2307/2214187

[B11] Block N. (1995). On a confusion about the function of consciousness. *Behav. Brain Sci.* 18 227–247. 10.1017/S0140525X00038188

[B12] Block N. (1996). “Mental paint and mental latex,” in *Philosophical Issues*, Vol. 7 ed. Villenueva E. (Northridge: Ridgeview Publishing Company).

[B13] Block N. (2003). “Mental paint,” in *Reflections and Replies: Essays on the Philosophy of Tyler Burge*, eds Hahn M. Ramberg B. (Cambridge, MA: MIT Press).

[B14] Block N. (2007). Consciousness, accessibility, and the mesh between psychology and neuroscience. *Behav. Brain Sci.* 30 481–538. 10.1017/S0140525X07002786 18366828

[B15] Boorse C. (1975). On the distinction between disease and illness. *Philos. Public Aff.* 5 49–68.

[B16] Boorse C. (1976). What a theory of mental health should be. *J. Theory Soc. Behav.* 6 61–84. 10.1111/j.1468-5914.1976.tb00359.x

[B17] Boorse C. (1977). Health as a theoretical concept. *Philos. Sci.* 44 542–573. 10.1086/288768

[B18] Boorse C. (1997). “A rebuttal on health,” in *What is Disease?*, eds Humber J. M. Almeder R. F. (Totowa, NJ: Humana Press).

[B19] Brentano F. (1874/1995). *Psychology from an Empirical Standpoint.* New York, NY: Routledge & Kegan Paul.

[B20] Bynum T. W. Moor J. H. (eds) (2003). *CyberPhilosophy: The Intersection of Philosophy and Computing.* Oxford: Basil Blackwell.

[B21] Campbell C. A. (1951). Is ‘freewill’ a pseudo-problem? *Mind* 60 441–465. 10.1093/mind/LX.240.441

[B22] Carruthers P. (2000). *Phenomenal Consciousness: A Naturalistic Theory.* Cambridge: Cambridge University Press.

[B23] Carter B. (1974). “Large number coincidences and the anthropic principle in cosmology,” in *Proceedings of the Confrontation of Cosmological Theories with Observational Data*, ed. Longair M. (Dordrecht: D. Reidel Publishing Co).

[B24] Chalmers D. J. (1995). Facing up to the problem of consciousness. *J. Conscious. Stud.* 2 200–219.

[B25] Chalmers D. J. (1996). *The Conscious Mind: In Search of a Fundamental Theory.* New York, NY: Oxford University Press.

[B26] Chalmers D. J. (2009). “The two-dimensional argument against materialism,” in *The Oxford Handbook of Philosophy of Mind*, eds McLaughlin B. P. Beckermann A. Walter S. (Oxford: Oxford University Press).

[B27] Chalmers D. J. (2010). *The Character of Consciousness.* Oxford: Oxford University Press.

[B28] Chemero A. (2009). *Radical Embodied Cognitive Science.* Cambridge, MA: MIT Press.

[B29] Chisholm R. M. (1964). *Human Freedom and the Self.* Lawrence, KS: University of Kansas.

[B30] Chomsky N. (1975). *Reflections on Language.* New York, NY: Pantheon.

[B31] Chomsky N. (1980). *Rules and Representations.* New York, NY: Columbia University Press.

[B32] Chomsky N. (1988). *Language and Problems of Knowledge: The Managua Lectures.* Cambridge, MA: MIT Press.

[B33] Chomsky N. (2000). *New Horizons in the Study of Language and Mind.* Cambridge: Cambridge University Press.

[B34] Clark A. (1997). *Being There: Putting Mind, Body, and World Together Again.* Cambridge, MA: MIT Press.

[B35] Clark A. (2001). Reasons, robots, and the extended mind. *Mind Lang.* 16 121–145. 10.1111/1468-0017.00162

[B36] Clark A. (2003). *Natural-Born Cyborgs: Minds, Technologies, and the Future of Human Intelligence.* New York, NY: Oxford University Press.

[B37] Clark A. (2008). *Supersizing the Mind: Embodiment, Action, and Cognitive Extension.* Oxford: Oxford University Press.

[B38] Clark A. Chalmers D. J. (1998). The extended mind. *Analysis* 58 7–19. 10.1093/analys/58.1.7

[B39] Clark A. Kiverstein J. Vierkant T. (eds) (2013). *Decomposing the Will.* Oxford: Oxford University Press.

[B40] Cohen M. A. Dennett D. C. (2011). Consciousness cannot be separated from function. *Trends Cogn. Sci.* 15 358–364. 10.1016/j.tics.2011.06.008 21807333

[B41] Cooper A. (forthcoming) Two directions for teleology: naturalism and idealism. *Synthese* 9. 10.1007/s11229-017-1364-5

[B42] Crane T. (1998a). Intentionality as the mark of the mental. *R. Inst. Philos. Suppl.* 43 229–251.

[B43] Crane T. (1998b). “Intentionality,” in *The Routledge Encyclopedia of Philosophy*, ed. Craig E. (Abingdon: Taylor and Francis).

[B44] Crane T. (2001). Intentional objects. *Ratio* 14 336–349. 10.1111/1467-9329.00168

[B45] Crane T. (2009). “Intentionalism,” in *The Oxford Handbook of Philosophy of Mind*, eds McLaughlin B. P. Beckermann A. Walter S. (Oxford: Oxford University Press).

[B46] Crane T. (2012). What is the problem of non-existence? *Philosophia* 40 417–434. 10.1007/s11406-011-9354-1

[B47] Crane T. (2013). *The Objects of Thought.* Oxford: Oxford University Press.

[B48] Crick F. Koch C. (1990). Towards a neurobiological theory of consciousness. *Semin. Neurosci.* 2 263–275.

[B49] Crick F. Koch C. (2003). A framework for consciousness. *Nat. Neurosci.* 6 119–126. 10.1038/nn0203-119 12555104

[B50] Crosson F. J. Sayre K. M. (eds) (1967). *Philosophy and Cybernetics: Essays Delivered to the Philosophic Institute for Artificial Intelligence at the University of Notre Dame.* Notre Dame: University of Notre Dame Press.

[B51] Darwin C. R. (1859). *On the Origin of Species by Means of Natural Selection, or the Preservation of Favoured Races in the Struggle for Life.* London: John Murray. PMC5184128 30164232

[B52] Davidson D. H. (1963). Actions, reasons and causes. *J. Philos.* 60 685–700. 10.2307/2023177

[B53] Davidson D. H. (1974). Belief and the basis of meaning. *Synthese* 27 309–323. 10.1007/BF00484597

[B54] Dennett D. C. (1988). “Quining qualia,” in *Consciousness in Contemporary Science*, eds Marcel A. J. Bisiach E. (Oxford: Oxford University Press).

[B55] Dennett D. C. (1991). *Consciousness Explained.* Boston, MA: Little, Brown & Company.

[B56] Dennett D. C. (1992). “The self as the center of narrative gravity,” in *Self and Consciousness: Multiple Perspectives*, eds Kessel F. Cole P. Johnson D. L. (Hillsdale, NJ: Lawrence Erlbaum).

[B57] Dennett D. C. (1993). The message is: there is no medium. *Philos. Phenomenol. Res.* 53 919–931. 10.2307/2108264

[B58] Dennett D. C. (1994). “Instead of qualia,” in *Consciousness in Philosophy and Cognitive Neuroscience*, eds Revonsuo A. Kamppinen M. (Hillsdale, NJ: Lawrence Erlbaum).

[B59] Dennett D. C. Kinsbourne M. (1992). Time and the observer: the where and when of consciousness in the brain. *Behav. Brain Sci.* 15 187–247. 10.1007/s00221-015-4231-y

[B60] D’Oro G. Sandis C. (eds) (2013). *Reasons and Causes: Causalism and Anti-Causalism in the Philosophy of Action.* London: Palgrave Macmillan.

[B61] Dretske F. I. (1988). *Explaining Behavior.* Cambridge, MA: MIT Press.

[B62] Dretske F. I. (1995). *Naturalizing the Mind.* Cambridge MA: MIT Press.

[B63] Dretske F. I. (1998). “Minds, machines, and money: what really explains behavior,” in *Human Action, Deliberation and Causation*, eds Bransen J. A. M. Cuypers S. E. (Dordrecht: Kluwer).

[B64] Dretske F. I. (2004). Change blindness. *Philos. Stud.* 120 1–18. 10.1023/B:PHIL.0000033749.19147.88

[B65] Dretske F. I. (2007). What change blindness teaches about consciousness. *Philosophical Perspectives* 21 215–220. 10.1111/j.1520-8583.2007.00126.x

[B66] Dretske F. I. (2015). “Supervenience and the causal explanation of behavior,” in *Qualia and Mental Causation in a Physical World: Themes from the Philosophy of Jaegwon Kim*, eds Horgan T. Sabatés M. Sosa D. (Cambridge: Cambridge University Press).

[B67] Eccles J. C. (1994). *How the Self Controls its Brain.* Berlin: Springer-Verlag 10.1007/978-3-642-49224-2

[B68] Ekstrom L. W. (2011). “Free will is not a mystery,” in *The Oxford Handbook of Free Will*, 2nd Edn, ed. Kane R. (Oxford: Oxford University Press).

[B69] Fahrenfort J. J. Lamme V. A. F. (2012). A true science of consciousness explains phenomenology: comment on Cohen and Dennett. *Trends Cogn. Sci.* 16 138–139. 10.1016/j.tics.2012.01.004 22300549

[B70] Fara M. (2008). Masked abilities and compatibilism. *Mind* 117 843–865. 10.1093/mind/fzn078

[B71] Farkas K. (2008a). *The Subject’s Point of View.* Oxford: Oxford University Press.

[B72] Farkas K. (2008b). Phenomenal intentionality without compromise. *Monist* 91 273–293.

[B73] Field H. (2001). *Truth and the Absence of Fact.* Oxford: Clarendon Press.

[B74] Flanagan O. (1991). *The Science of the Mind*, 2nd Edn Cambridge, MA: MIT Press.

[B75] Flanagan O. (1992). *Consciousness Reconsidered.* Cambridge, MA: MIT Press.

[B76] Flanagan O. (1998). *The Nature of Consciousness.* Cambridge MA: MIT Press.

[B77] Fodor J. A. (1975). *The Language of Thought.* Cambridge, MA: Harvard University Press.

[B78] Fodor J. A. (1987). *Psychosemantics: The Problem of Meaning in the Philosophy of Mind.* Cambridge, MA: MIT Press.

[B79] Fodor J. A. (1989). Making mind matter more. *Philos. Top.* 17 59–79. 10.5840/philtopics198917112

[B80] Fodor J. A. (2008). *LOT 2: The Language of Thought Revisited.* Oxford: Oxford University Press.

[B81] Foster J. A. (1989). “A defense of dualism,” in *The Case for Dualism*, eds Smythies J. Beloff J. (Charlottesville, VA: University of Virginia Press).

[B82] Foster J. A. (1996). *The Immaterial Self: A Defence of the Cartesian Dualist Conception of Mind.* London: Routledge.

[B83] Gallagher S. (2000a). Philosophical conceptions of the self: implications for cognitive science. *Trends Cogn. Sci.* 4 14–21. 10637618 10.1016/s1364-6613(99)01417-5

[B84] Gallagher S. (2000b). “Self-reference and schizophrenia: a cognitive model of immunity to error through misidentification,” in *Exploring the Self: Philosophical and Psychopathological Perspectives on Self-Experience*, ed. Dan Zahavi (Amsterdam: John Benjamins).

[B85] Gallagher S. (2008). “Self-agency and mental causality,” in *Philosophical Issues in Psychiatry: Explanation, Phenomenology, and Nosology*, eds Kendler K. S. Parnas J. (Baltimore, MD: Johns Hopkins University Press).

[B86] Gallagher S. (2015). Relations between agency and ownership in the case of schizophrenic thought insertion. *Rev. Philos. Psychol.* 6 865–879. 10.1007/s13164-014-0222-3

[B87] Goetz S. (2008). *Freedom, Teleology, and Evil.* Norfolk, VA: Continuum.

[B88] Goff P. (2017). *Consciousness and Fundamental Reality.* Oxford: Oxford University Press.

[B89] Hameroff S. R. Penrose R. (1996). Conscious events as orchestrated spacetime selections. *J. Conscious. Stud.* 3 36–53.

[B90] Harman G. (1990). “The intrinsic quality of experience,” in *Philosophical Perspectives*, Vol. 4 ed. Tomberlin J. (Atascadero, CA: Ridgeview Publishing).

[B91] Harris S. (2012). *Free Will.* New York, NY: Free Press.

[B92] Hempel C. G. (1969). “Reduction: ontological and linguistic facets,” in *Philosophy, Science, and Method: Essays in Honor of Ernest Nagel*, eds Morgenbesser S. Suppes P. White M. (New York, NY: St. Martins Press).

[B93] Hilbert D. R. Kalderon M. E. (2000). “Color and the inverted spectrum,” in *Color Perception: Philosophical, Psychological, Artistic, and Computational Perspectives*, ed. Davis S. (Oxford: Oxford University Press).

[B94] Horgan T. Kriegel U. (2008). Phenomenal intentionality meets the extended mind. *Monist* 91 347–373. 10.5840/monist20089128

[B95] Horgan T. Tienson J. (2002). “The intentionality of phenomenology and the phenomenology of intentionality,” in *Philosophy of Mind: Classical and Contemporary Readings*, ed. Chalmers D. J. (New York, NY: Oxford University Press).

[B96] Hume D. (1738). *A Treatise of Human Nature: An Attempt to Introduce the Experimental Method of Reasoning into Moral Subjects.* London: John Noon.

[B97] Husserl E. (1901). *Logische Untersuchungen. Zweite Teil: Untersuchungen zur Phänomenologie und Theorie der Erkenntnis.* Halle: Max Niemeyer.

[B98] Huxley T. S. (1869). *The Elements of Physiology and Hygiene: A Text-Book for Educational Institutions.* New York, NY: D. Appleton.

[B99] Jackson F. (1982). Epiphenomenal qualia. *Philos. Q.* 32 127–136. 10.2307/2960077

[B100] Jackson F. (1986). What Mary didn’t know. *J. Philos.* 83 291–295. 10.2307/2026143

[B101] Jacob P. (2014). “Intentionality,” in *The Stanford Encyclopedia of Philosophy (Winter 2014 Edition)*, ed. Zalta E. N. Available at: http://plato.stanford.edu/archives/win2013/entries/intentionality/

[B102] Johnson M. R. (2005). *Aristotle on Teleology.* Oxford: Clarendon Press.

[B103] Kane R. (1999). Responsibility, luck, and chance: reflections on free will and indeterminism. *J. Philos.* 96 217–240. 10.5840/jphil199996537

[B104] Kim J. (1971). Materialism and the criteria of the mental. *Synthese* 22 323–345. 10.1007/BF00413431

[B105] Kim J. (1989). Mechanism, purpose and explanatory exclusion. *Philos. Perspect.* 3 77–108. 10.1016/j.shpsc.2011.05.016

[B106] Kim J. (1998). *Mind in a Physical World: An Essay on the Mind-Body Problem and Mental Causation.* Cambridge, MA: MIT Press.

[B107] Kim J. (2005). *Physicalism, or Something Near Enough.* Princeton, NJ: Princeton University Press.

[B108] Kim J. (2007). “Causation and mental causation,” in *Contemporary Debates in Philosophy of Mind*, eds McLaughlin B. Cohen J. (Oxford: Basil Blackwell).

[B109] Kim J. (2011). *Philosophy of Mind*, 3rd Edn. Boulder, CO: Westview Press.

[B110] Kirk R. Squires J. E. R. (1974). Zombies v. materialists. *Proc. Aristotelian Soc.* 48 135–164. 10.1093/aristoteliansupp/48.1.135

[B111] Koch C. (2004). *The Quest for Consciousness: A Neurobiological Approach.* Englewood, CO: Roberts & Company.

[B112] Koch C. (2012). *Consciousness: Confessions of a Romantic Reductionist.* Cambridge, MA: MIT Press.

[B113] Kriegel U. (2010). Intentionality and normativity. *Philos. Issues* 20 185–208. 10.1111/j.1533-6077.2010.00182.x

[B114] Kriegel U. (2011). *Sources of Intentionality.* Oxford: Oxford University Press.

[B115] Kripke S. A. (1982). *Wittgenstein on Rules and Private Language.* Oxford: Basil Blackwell.

[B116] Lamme V. A. F. (2006). Toward a true neural stance on consciousness. *Trends Cogn. Sci.* 10 494–501. 10.1016/j.tics.2006.09.001 16997611

[B117] Lamme V. A. F. (2010). How neuroscience will change our view on consciousness. *Cogn. Neurosci.* 1 204–220. 10.1080/17588921003731586 24168336

[B118] Lau H. C. Rogers R. D. Ramnani N. Passingham R. E. (2004). Willed action and attention to the selection of action. *Neuroimage* 21 1407–1415. 10.1016/j.neuroimage.2003.10.034 15050566

[B119] Laughlin C. D. McManus J. d’Aquili E. (1990). *Brain, Symbol and Experience: Toward a Neurophenomenology of Consciousness.* Boston, MA: New Science Library.

[B120] Lehrer K. (1968). Cans without ifs. *Analysis* 29 29–32. 10.1093/analys/29.1.29

[B121] Leuenberger S. (2008). “Ceteris absentibus physicalism,” in *Oxford Studies in Metaphysics*, Vol. 4 ed. Zimmerman D. (Oxford: Oxford University Press).

[B122] Leunissen M. (2010). *Explanation and Teleology in Aristotle’s Science of Nature.* New York, NY: Cambridge University Press.

[B123] Levine J. (1983). Materialism and qualia: the explanatory gap. *Pac. Philos. Q.* 64 354–361.

[B124] Levison A. B. (1983). An epistemic criterion of the mental. *Can. J. Philos.* 13 389–407. 10.1111/j.1365-2753.2012.01914.x

[B125] Levy N. (2005). Libet’s impossible demand. *J. Conscious. Stud.* 12 67–76. 10.1016/j.encep.2014.01.001

[B126] Lewis D. C. (1981). Are we free to break the laws? *Theoria* 47 113–121. 10.1111/j.1755-2567.1981.tb00473.x

[B127] Libet B. (1985). Unconscious cerebral initiative and the role of conscious will in voluntary action. *Behav. Brain Sci.* 85 529–566. 10.1017/S0140525X00044903

[B128] Libet B. (1994). A testable field theory of mind-brain interaction. *J. Conscious. Stud.* 1 119–126.

[B129] Libet B. (2002). “Do we have free will?,” in *The Oxford Companion to Free Will*, ed. Kane R. (Oxford: Oxford University Press).

[B130] Libet B. (2003). Can conscious experience affect brain activity? *J. Conscious. Stud.* 10 24–28.

[B131] Libet B. (2004). *Mind Time: The Temporal Factor in Consciousness.* Cambridge, MA: Harvard University Press.

[B132] Libet B. (2006). Reflections on the interaction of the mind and the brain. *Prog. Neurobiol.* 78 322–326. 10.1016/j.pneurobio.2006.02.003 16675090

[B133] Locke J. (1689). *An Essay Concerning Humane Understanding.* London: Thomas Basset.

[B134] Logan R. K. (2007). *The Extended Mind: The Emergence of Language, the Human Mind and Culture.* Toronto, ON: University of Toronto Press.

[B135] Lycan W. (1987). *Consciousness.* Cambridge, MA: MIT Press.

[B136] Lycan W. (1996). *Consciousness and Experience.* Cambridge, MA: MIT Press.

[B137] MacDonald G. Papineau D. (eds) (2006). *Teleosemantics: New Philosophical Essays.* Oxford: Oxford University Press.

[B138] Malafouris L. (2010). “Knapping intentions and the marks of the mental,” in *The Cognitive Life of Things: Recasting the Boundaries of the Mind*, eds Malafouris L. Renfrew C. (Cambridge: McDonald Institute Monographs).

[B139] Malafouris L. (2013). Mind into matter: where do you end, and the outside world begin? *New Sci.* 219 28–29. 10.1016/S0262-4079(13)62191-0

[B140] Mayr E. W. (1974). Teleological and teleonomic. A new analysis. *Boston Stud. Philos. Sci.* 14 91–117. 10.1007/978-94-010-2128-9_6

[B141] Mayr E. W. (1988). *Toward a New Philosophy of Biology: Observations of an Evolutionist.* Cambridge, MA: Harvard University Press.

[B142] Mayr E. W. (1992). The idea of teleology. *J. Hist. Ideas* 53 117–135. 10.1016/j.shpsc.2015.07.003

[B143] Mayr E. W. (1998). The multiple meanings of teleological. *Hist. Philos. Life Sci.* 20 35–40.

[B144] Mayr E. W. (2004). *What Makes Biology Unique? Considerations on the Autonomy of a Scientific Discipline.* Cambridge: Cambridge University Press.

[B145] McDowell J. (1994). *Mind and World.* Cambridge, MA: Harvard University Press.

[B146] McGinn C. (1989a). Can we solve the mind-body problem? *Mind* 98 349–366. 10.1093/mind/XCVIII.391.349

[B147] McGinn C. (1989b). *Mental Content.* Oxford: Oxford University Press.

[B148] McGinn C. (1991). *The Problem of Consciousness.* Oxford: Basil Blackwell.

[B149] McGinn C. (1995). “Consciousness and space,” in *Conscious Experience*, ed. Metzinger T. (Paderborn: Ferdinand Schöningh).

[B150] Meinong A. R. V. H. (1899). Über gegenstände höherer ordnung und deren verhältnis zur inneren wahrnehmung. *Z. Psychol. Physiol. Sinnesor.* 21 182–272.

[B151] Mele A. R. (2014a). *A Dialogue on Free Will and Science.* Oxford: Oxford University Press.

[B152] Mele A. R. (2014b). *Free: Why Science Hasn’t Disproved Free Will.* Oxford: Oxford University Press.

[B153] Menary R. (2007). *Cognitive Integration: Mind and Cognition Unbounded.* New York, NY: Palgrave Macmillan.

[B154] Menary R. (ed.) (2010). *The Extended Mind.* Cambridge, MA: MIT Press.

[B155] Millikan R. G. (1984). *Language, Thought and Other Biological Categories.* Cambridge, MA: MIT Press.

[B156] Millikan R. G. (1993). *White Queen Psychology and Other Essays.* Cambridge, MA: MIT Press.

[B157] Millikan R. G. (2000). *On Clear and Confused Ideas.* Cambridge: Cambridge University Press.

[B158] Millikan R. G. (2004). *Varieties of Meaning.* Cambridge, MA: MIT Press.

[B159] Millikan R. G. (2005). *Language: A Biological Model.* Oxford: Oxford University Press.

[B160] Montero B. (1999). The body problem. *Noûs* 33 183–200. 10.1111/0029-4624.00149

[B161] Moore G. E. (1903). *Principia Ethica.* Cambridge: Cambridge University Press.

[B162] Mulhall S. (2007). *Wittgenstein’s Private Language: Grammar, Nonsense, and Imagination in Philosophical Investigations.* Oxford: Clarendon Press.

[B163] Nagel T. (1974). What is it like to be a bat? *Philos. Rev.* 83 435–456. 10.2307/2183914

[B164] Nagel T. (1979). “Panpsychism,” in *Mortal Questions*, ed. Nagel T. (Cambridge: Cambridge University Press).

[B165] Nagel T. (2012). *Mind and Cosmos: Why the Materialist Neo-Darwinian Conception of Nature is Almost Certainly False.* Oxford: Oxford University Press.

[B166] Nahmias E. (2002). When consciousness matters: a critical review of Daniel Wegner’s The Illusion of Conscious Will. *Philos. Psychol.* 15 527–541. 10.1080/0951508021000042049

[B167] Nahmias E. (2011). “Intuitions about free will, determinism, and bypassing,” in *The Oxford Handbook of Free Will*, 2nd Edn, ed. Kane R. (New York, NY: Oxford University Press).

[B168] Neander K. (2017). *A Mark of the Mental: In Defense of Informational Teleosemantics.* Cambridge, MA: MIT Press.

[B169] Papineau D. (1984). Representation and Explanation. *Philos. Sci.* 51 550–572. 10.1086/289205

[B170] Papineau D. (1987). *Reality and Representation.* Oxford: Basil Blackwell.

[B171] Papineau D. (1993). *Philosophical Naturalism.* Oxford: Basil Blackwell.

[B172] Papineau D. (2002). *Thinking about Consciousness.* Oxford: Oxford University Press.

[B173] Pautz A. (2013). “Does phenomenology ground mental content?,” in *Phenomenal Intentionality*, ed. Kriegel U. (Oxford: Oxford University Press).

[B174] Peacocke C. (1983). *Sense and Content: Experience, Thought and their Relations.* Oxford: Oxford University Press.

[B175] Penrose R. (1994). *Shadows of the Mind: A Search for the Missing Science of Consciousness.* Oxford: Oxford University Press.

[B176] Pereboom D. Caruso G. D. (2002). “Hard-incompatibilist existentialism: neuroscience, punishment, and meaning in life,” in *Neuroexistentialism: Meaning, Morals, and Purpose in the Age of Neuroscience*, eds Caruso G. D. Flanagan O. (Oxford: Oxford University Press).

[B177] Pernu T. K. (2011). Minding matter: how not to argue for the causal efficacy of the mental. *Rev. Neurosci.* 22 483–507. 10.1515/RNS.2011.043 21967516

[B178] Pernu T. K. (2013). The principle of causal exclusion does not make sense. *Philos. Forum* 44 89–95. 10.1111/phil.12003

[B179] Pinker S. (1997). *How the Mind Works.* New York, NY: W. W. Norton & Company.

[B180] Pitt D. (2004). The phenomenology of cognition, or, what is it like to think that P? *Philos. Phenomenol. Res.* 69 1–36. 10.1016/j.alcohol.2014.06.002

[B181] Place U. T. (1996). Intentionality as the mark of the dispositional. *Dialectica* 50 91–120. 10.1111/j.1746-8361.1996.tb00001.x

[B182] Preston J. Bishop M. (eds) (2002). *Views into the Chinese Room: New Essays on Searle and Artificial Intelligence.* New York, NY: Oxford University Press.

[B183] Price C. S. (2001). *Functions in Mind: A Theory of Intentional Content.* Oxford: Clarendon Press.

[B184] Priest G. (2005). *Towards Non-Being: The Logic and Metaphysics of Intentionality.* Oxford: Oxford University Press.

[B185] Prinz W. (2003). Emerging selves: representational foundations of subjectivity. *Conscious. Cogn.* 12 515–528. 10.1016/S1053-8100(03)00053-9 14656495

[B186] Robinson H. M. (1982). *Matter and Sense: A Critique of Contemporary Materialism.* Cambridge: Cambridge University Press.

[B187] Rorty R. (1970). Incorrigibility as the mark of the mental. *J. Philos.* 67 399–424. 10.2307/2024002

[B188] Rorty R. (1979). *Philosophy and the Mirror of Nature.* Oxford: Basil Blackwell.

[B189] Rosenberg G. (2004). *A Place for Consciousness: Probing the Deep Structure of the Natural World.* New York, NY: Oxford University Press.

[B190] Sayre K. M. (1976). *Cybernetics and the Philosophy of Mind.* Atlantic Highlands, NJ: Humanities Press.

[B191] Schlick M. (1930). *Fragen der Ethik.* Vienna: Verlag von Julius Springer.

[B192] Schueler G. F. (2003). *Reasons and Purposes: Human Rationality and the Teleological Explanation of Action.* Oxford: Oxford University Press.

[B193] Schwartz J. M. Stapp H. P. Beauregard M. (2005). Quantum theory in neuroscience and psychology: a neurophysical model of mind/brain interaction. *Philos. Trans. R. Soc. B* 360 1309–1327. 10.1098/rstb.2004.1598 PMC1569494 16147524

[B194] Seager W. E. (1995). Consciousness, information, and panpsychism. *J. Conscious. Stud.* 2 272–288.

[B195] Seager W. E. (2006). The ’intrinsic nature’ argument for panpsychism. *J. Conscious. Stud.* 13 129–145.

[B196] Searle J. S. (1980). Minds, brains and programs. *Behav. Brain Sci.* 3 417–457. 10.1017/S0140525X00005756

[B197] Sehon S. R. (2005). *Teleological Realism: Mind, Agency and Explanation.* Cambridge, MA: MIT Press.

[B198] Sehon S. R. (2016). *Free Will and Action Explanation: A Non-Causal, Compatibilist Account.* Oxford: Oxford University Press.

[B199] Seth A. K. (2016). “The real problem,” in *Aeon*, 02 November, 2016.

[B200] Setiya K. (2011). Reasons and causes. *Eur. J. Philos.* 19 129–157. 10.1111/j.1468-0378.2009.00378.x

[B201] Shepherd J. (2012). Free will and consciousness: experimental studies. *Conscious. Cogn.* 21 915–927. 10.1016/j.concog.2012.03.004 22480780

[B202] Shepherd J. (2015). Consciousness, free will, and moral responsibility: taking the folk seriously. *Philos. Psychol.* 28 929–946. 10.1080/09515089.2014.962018 26692640 PMC4647831

[B203] Shepherd J. (2016). Conscious action/zombie action. *Noûs* 50 219–244. 10.1111/nous.12086 PMC5032892 27667859

[B204] Shoemaker S. (1975). Functionalism and qualia. *Philos. Stud.* 27 291–315. 10.1007/BF01225748

[B205] Shoemaker S. (1982). The inverted spectrum. *J. Philos.* 79 357–381. 10.2307/2026213

[B206] Shoemaker S. (1996). *The First-Person Perspective and Other Essays.* Cambridge: Cambridge University Press.

[B207] Siewert C. P. (1998). *The Significance of Consciousness.* Princeton, NJ: Princeton University Press.

[B208] Smart J. J. C. (1961). Free will, praise and blame. *Mind* 70 291–306. 10.1093/mind/LXX.279.291

[B209] Smith M. A. (1997). “A theory of freedom and responsibility,” in *Ethics and Practical Reason*, eds Cullity G. Gaut B. (Oxford: Oxford University Press).

[B210] Smith M. A. (2003). “Rational capacities, or: how to distinguish recklessness, weakness, and compulsion,” in *Weakness of Will and Practical Irrationality*, eds Stroud S. Tappolet C. (Oxford: Clarendon Press).

[B211] Soon C. S. Brass M. Heinze H.-J. Haynes J.-D. (2008). Unconscious determinants of free decisions in the human brain. *Nat. Neurosci.* 11 543–545. 10.1038/nn.2112 18408715

[B212] Stapp H. P. (1999). Attention, intention, and will in quantum physics. *J. Conscious. Stud.* 6 143–164.

[B213] Stapp H. P. (2009). *Mind, Matter and Quantum Mechanics*, 3rd Edn. Berlin: Springer-Verlag. 10.1007/978-3-540-89654-8

[B214] Sterelny K. (1990). *The Representational Theory of Mind: An Introduction.* Cambridge, MA: Basil Blackwell.

[B215] Stich S. P. (1983). *From Folk Psychology to Cognitive Science.* Cambridge, MA: MIT Press.

[B216] Strawson G. (2006). Realistic monism: why physicalism entails panpsychism. *J. Conscious. Stud.* 13 3–31.

[B217] Suppes P. (1970). *A Probabilistic Theory of Causation.* Helsinki: Societas Philosophica Fennica.

[B218] Székely L. (2015). *Louis C.K.: Live at the Comedy Store.* New York, NY: Louisck.com.

[B219] Tartaglia J. (2008). Intentionality, consciousness, and the mark of the mental: Rorty’s challenge. *Monist* 91 324–346. 10.5840/monist20089127

[B220] Tegmark M. (2000). Importance of quantum decoherence in brain processes. *Phys. Rev. E* 61 4194–4206. 10.1103/PhysRevE.61.4194 11088215

[B221] Tononi G. (2008). Consciousness as integrated information: a provisional manifesto. *Biol. Bull.* 215 216–242. 10.2307/25470707 19098144

[B222] Tye M. (1995). *Ten Problems of Consciousness.* Cambridge, MA: MIT Press.

[B223] Tye M. (2000). *Consciousness, Color, and Content.* Cambridge, MA: MIT Press.

[B224] Van Gulick R. (2016). *Consciousness.* Available at: http://plato.stanford.edu/archives/win2016/entries/consciousness/

[B225] van Inwagen P. (1983). *An Essay on Free Will.* Oxford: Oxford University Press.

[B226] van Inwagen P. (2000). Free will remains a mystery. *Philos. Perspect.* 14 1–20.

[B227] van Inwagen P. (2004). Freedom to break the laws. *Midwest Stud. Philos.* 28 336–350. 10.1111/j.1475-4975.2004.00099.x

[B228] van Inwagen P. (2008). How to think about the problem of free will. *J. Ethics* 12 327–341. 10.1007/s10892-008-9038-7

[B229] Varela F. J. (1996). Neurophenomenology: a methodological remedy to the hard problem. *J. Conscious. Stud.* 3 330–349.

[B230] Vierkant T. Kiverstein J. Clark A. (2013). “Decomposing the will: meeting the zombie challenge,” in *Decomposing the Will*, eds Clark A. Kiverstein J. Vierkant T. (Oxford: Oxford University Press).

[B231] Vihvelin K. (2000). Libertarian compatibilism. *Philos. Perspect.* 14 139–166. 10.1016/j.jaging.2016.02.003

[B232] Vihvelin K. (2004). Free will demystified: a dispositional account. *Philos. Top.* 32 427–450. 10.5840/philtopics2004321/211

[B233] Vihvelin K. (2011). “How to think about the free will/determinism problem,” in *Carving Nature at Its Joints: Natural Kinds in Metaphysics and Science*, eds Campbell J. K.’Rourke O M. Slater M. H. (Cambridge, MA: MIT Press).

[B234] Vihvelin K. (2013). *Causes, Laws, and Free Will: Why Determinism Doesn’t Matter.* New York, NY: Oxford University Press.

[B235] Voltolini A. (2013). The mark of the mental. *Phenomenol. Mind* 4 124–136.

[B236] von Wright G. H. (1971). *Explanation and Understanding.* Ithaca, NY: Cornell University Press.

[B237] Wakefield J. C. (1992). The concept of mental disorder. *American Psychologist* 47 373–388. 10.1037/0003-066X.47.3.373 1562108

[B238] Wakefield J. C. (1997a). Diagnosing DSM-IV, part 1: DSM-IV and the concept of disorder. *Behav. Res. Therapy* 35 633–649. 10.1016/S0005-7967(97)00018-1 9193127

[B239] Wakefield J. C. (1997b). Normal inability versus pathological inability. *Clin. Psychol.* 4 249–258.

[B240] Waller R. R. (2012). Beyond button presses: the neuroscience of free and morally appraisable actions. *Monist* 95 441–462. 10.5840/monist201295323

[B241] Watson G. (1987). Free action and free will. *Mind* 96 145–172. 10.1093/mind/XCVI.382.145

[B242] Wegner D. M. (2002). *The Illusion of Conscious Will.* Cambridge, MA: MIT Press.

[B243] Wicken J. S. (1981). Causal explanations in classical and statistical thermodynamics. *Philos. Sci.* 48 65–77. 10.1086/288977

[B244] Wiener N. (1948). *Cybernetics, or, Control and Communication in the Animal and the Machine.* Cambridge, MA: Technology Press.

[B245] Wiener N. (1950). *The Human Use of Human Beings: Cybernetics and Society.* Boston, MA: Houghton Mifflin.

[B246] Wilson R. A. (2005). Collective memory, group minds, and the extended mind thesis. *Cogn. Process.* 6 227–236. 10.1007/s10339-005-0012-z 18239951

[B247] Wittgenstein L. J. J. (1953). *Philosophical Investigations.* Oxford: Basil Blackwell.

[B248] Zangwill N. (2005). The normativity of the mental. *Philos. Explor.* 8 1–20. 10.1080/1386979042000336126

[B249] Zangwill N. (2010). Normativity and the metaphysics of mind. *Austr. J. Philos.* 88 21–39. 10.1080/00048400902739610

